# Phosphorylation of TRF2 promotes its interaction with TIN2 and regulates DNA damage response at telomeres

**DOI:** 10.1093/nar/gkac1269

**Published:** 2023-01-18

**Authors:** Radka Storchova, Matous Palek, Natalie Palkova, Pavel Veverka, Tomas Brom, Ctirad Hofr, Libor Macurek

**Affiliations:** Cancer Cell Biology, Institute of Molecular Genetics of the Czech Academy of Sciences, Prague CZ-14220, Czech Republic; Cancer Cell Biology, Institute of Molecular Genetics of the Czech Academy of Sciences, Prague CZ-14220, Czech Republic; Cancer Cell Biology, Institute of Molecular Genetics of the Czech Academy of Sciences, Prague CZ-14220, Czech Republic; LifeB, Functional Genomics and Proteomics, National Centre for Biomolecular Research, Faculty of Science, Masaryk University, Brno CZ-62500, Czech Republic; LifeB, Functional Genomics and Proteomics, National Centre for Biomolecular Research, Faculty of Science, Masaryk University, Brno CZ-62500, Czech Republic; LifeB, Functional Genomics and Proteomics, National Centre for Biomolecular Research, Faculty of Science, Masaryk University, Brno CZ-62500, Czech Republic; Cancer Cell Biology, Institute of Molecular Genetics of the Czech Academy of Sciences, Prague CZ-14220, Czech Republic

## Abstract

Protein phosphatase magnesium-dependent 1 delta (PPM1D) terminates the cell cycle checkpoint by dephosphorylating the tumour suppressor protein p53. By targeting additional substrates at chromatin, PPM1D contributes to the control of DNA damage response and DNA repair. Using proximity biotinylation followed by proteomic analysis, we identified a novel interaction between PPM1D and the shelterin complex that protects telomeric DNA. In addition, confocal microscopy revealed that endogenous PPM1D localises at telomeres. Further, we found that ATR phosphorylated TRF2 at S410 after induction of DNA double strand breaks at telomeres and this modification increased after inhibition or loss of PPM1D. TRF2 phosphorylation stimulated its interaction with TIN2 both *in vitro* and at telomeres. Conversely, induced expression of PPM1D impaired localisation of TIN2 and TPP1 at telomeres. Finally, recruitment of the DNA repair factor 53BP1 to the telomeric breaks was strongly reduced after inhibition of PPM1D and was rescued by the expression of TRF2-S410A mutant. Our results suggest that TRF2 phosphorylation promotes the association of TIN2 within the shelterin complex and regulates DNA repair at telomeres.

## INTRODUCTION

Genome instability is one of the hallmarks of cancer cells (1). DNA damage response driven by Ataxia telangiectasia mutated (ATM) and Ataxia telangiectasia and Rad3-related protein (ATR) kinases represents a surveillance mechanism that protects genome integrity by orchestrating a temporal cell cycle arrest and DNA repair (2–4). DNA double strand breaks (DSBs) are repaired either by non-homologous end joining (NHEJ) or by homologous recombination (HR). Protein phosphatase magnesium-dependent 1 delta (PPM1D, also known as WIP1) promotes recovery from the G2 checkpoint by counteracting activities of the tumour suppressor p53 and KRAB-interacting protein 1 (KAP1) (5,6). In addition, PPM1D terminates DNA damage response by directly targeting ATM, histone H2AX, BRCA1 and other proteins at the chromatin flanking the DNA lesions (7–10). Amplification of the *PPM1D* locus or gain-of-function mutations in the last exon of PPM1D have been reported to promote tumorigenesis by inhibiting p53 pathway and are commonly found in various solid tumours and haematological malignancies (11–14).

Although essential for preventing global genome instability, DNA repair at the ends of chromosomes needs to be actively suppressed to prevent the fusion of telomeric DNA (15). Integrity of the telomeres is protected by the shelterin complex comprising of telomeric repeat-binding factor 1 (TRF1), telomeric repeat-binding factor 2 (TRF2), TRF2-interacting telomeric protein 1 (TERF2IP; further referred to as RAP1), TRF1-interacting nuclear protein 2 (TIN2; also known as TINF2), protection of telomeres protein 1 (POT1), and Adrenocortical dysplasia protein homolog (ACD, hereafter referred to as TPP1) (16). TRF1 and TRF2 form homodimers through the TRFH domains, and they bind the TTAGGG repeats in the double-stranded telomeric DNA through their C-terminal Myb domains (17). In addition, the N-terminal basic domain of TRF2 can bind branched DNA structures and the double stranded DNA also wraps around the TRFH domain of TRF2 (18–20). The heterodimer comprising of TPP1 and POT1 associates with the single-stranded DNA through two oligonucleotide/oligosaccharide-binding (OB) folds of POT1 (21,22). In addition, TPP1 also promotes the recruitment of the telomerase (23). TIN2 bridges the TRF1 and TRF2 homodimers with TPP1 and prevents activation of ATR by stabilizing TPP1-POT1 at telomeric ssDNA (24–26). Similarly, TIN2 promotes TRF2 binding to telomeres thus protecting telomeric DNA from uncapping and from activation of ATM (26–29). Structural studies have revealed that TIN2 interacts with the TRFH domains of TRF1 and TRF2, and with a short motif between the residues 392–408 of TRF2 (hereafter referred to as a TIN2-binding motif, TBM) (30,31). Due to its unique DNA-binding ability, TRF2 promotes the folding of the telomeric DNA into a lasso-like structure referred to as a t-loop that prevents activation of ATM (15,32,33). In addition, the basic domain of TRF2 has been reported to prevent unwinding of the t-loops whereas recruitment of the Regulator of telomere elongation helicase 1 (RTEL1) by TRF2 promotes telomere unwinding during the replication (20,34,35). Loss of TRF2 leads to exposure of the DNA end, causing activation of ATM followed by ubiquitination-dependent recruitment of 53BP1 (forming nuclear patches termed Telomere dysfunction-Induced Foci (TIFs)) and subsequent fusion of telomeres by NHEJ (36–38). In contrast to TRF2, TRF1 is required for replication of the telomeric DNA and its loss leads to telomeric fragility (39). Single-molecule imaging revealed the ability of TIN2 and TRF2 to compact the telomeric DNA *in vitro*; however, the importance of DNA de-compaction for DNA repair at telomeres still remains unclear (40–43).

Here, we aimed to identify new substrates of PPM1D at chromatin. Using proximity biotinylation assay and immunoprecipitation, we identified the shelterin complex as a major interacting partner of PPM1D in human cells. Confocal microscopy confirmed a close association between PPM1D and shelterin at telomeres in various cell types. Since PPM1D directly interacted with TRF2 *in vitro*, we evaluated the ability of PPM1D to dephosphorylate TRF2 in cells. We found that ATR phosphorylated TRF2 at S410 upon CRISPR Cas9-mediated induction of DNA breaks at telomeres. Inhibition or loss of PPM1D significantly increased the level of TRF2-S410 phosphorylation. In addition, PPM1D dephosphorylated TRF2 *in vitro*. Importantly, increased phosphorylation of TRF2-S410 in cells treated with PPM1D inhibitor promoted the association of TIN2 with the damaged telomeres and prevented recruitment of the DNA repair factor 53BP1. Inversely, the expression of a non-phosphorylatable mutant TRF2-S410A rescued the recruitment of 53BP1 to DSBs at telomeres in cells treated with PPM1D inhibitor. Furthermore, overexpression of PPM1D impeded with assembly of the shelterin at telomeres and promoted telomeric fusions. We conclude that ATR and PPM1D control the binding of TIN2 at telomeres by inversely regulating the phosphorylation of TRF2 at S410.

## MATERIALS AND METHODS

### Cells

Human hTERT-immortalized RPE1 cells (here referred to as RPE), HEK293, human breast adenocarcinoma MCF7 or human osteosarcoma U2OS cells were grown in DMEM supplemented with 6% FBS (Gibco), Penicillin and Streptomycin. U2OS-PPM1D-KO cells with a knock-out of PPM1D were described previously (44). HeLa cells with doxycycline-inducible knock-down of TRF2 were described previously (45). HeLa-shTRF2 cells were transfected by pEGFP-TRF2 or pEGFP-TRF2-S410A and selected with geneticin followed by single cell clone expansion. RPE1 cells transfected with pCW57-GFP-P2A-PPM1D-A380 plasmid were selected by geneticin for 3 weeks followed by single clone expansion and expression of the catalytic domain of PPM1D was induced by doxycycline. All cells were regularly tested for mycoplasma infection using MycoAlert kit (Lonza). Plasmid DNA transfection was performed using polyethylenimine in ratio 1:6. Stable cell lines were generated by transfection of HEK293 cells with plasmid pBIOID2-HA or pBIOID2-PPM1D-D314A followed by 3 weeks selection with geneticin and expansion of single cell clones. Silencer Select siRNAs were transfected using RNAiMAX (both Thermo Scientific) at final concentration 5 nM and cells were analyzed after 2 days. Alternatively, two subsequent rounds of siRNA transfection were performed and cells were analyzed after 4 days. Expression of Cas9 was induced in iCut-RPE1 cells by overnight treatment with doxycycline and Shield-1 (1 μM, Aobious) and telomeric DNA damage was generated by transfection of the synthetic sgRNA TTAGGGTTAGGGTTAGGGTT (Sigma) as described previously (46,47). sgRNA was transfected by Lipofectamine RNAiMAX (Thermofisher) at final concentration 5 nM.

### Plasmids

Coding sequence of human TRF2 was PCR amplified from pLPC-NMyc-TRF2 (Addgene ID: 16066) (48) and inserted in frame into pEGFP plasmid. Mutagenesis of TRF2 was performed using PCR amplification followed by ligation of DNA fragments into pEGFP backbone by Gibson assembly kit (NEB). Correct mutagenesis was confirmed by sequencing. Numbering of the human TRF2 residues is based on reference sequence NP_005643. Phosphatase dead mutant PPM1D-D314A was cloned in frame into MCS-BioID2-HA (Addgene ID:74224). Constructs pEJS477-pHAGE-TO-Spy-dCas9-3Xm Cherry-SgRNA-Telomere-All-in-one (Addgene ID:85717) and pEJS469-pLK.O1-SpyS gRNA-DTS13-Telomere (Addgene ID: 85715) were used for visualization of telomeres. DNA double strand breaks at telomeres were induced by transfecting cells with pSpCas9(BB)-2A-GFP (PX458, Addgene ID:48138) containing the telomeric sgRNA, whereas the empty plasmid served as a negative control. DNA fragments corresponding to the full length human PPM1D, its deletion mutants lacking the Pro loop (ΔPro loop) or B loop (ΔB loop), fragment coding for unstructured C-terminal region (amino acids 370–605, CT) or fragment coding the catalytic domain (amino acids 1–380, A380) were ligated in frame into pEGFP or in pCW57-GFP-2A-MCS (Addgene ID: 71783) plasmids.

### Antibodies and reagents

The following antibodies were used in this study: TRF2 (ab108997, for WB), TIN2 (ab197894, for WB) from Abcam; TRF2 (NB110-57130, for IF), TIN2 (NBP2-55709, for IF), RAP1 (NBP1-82433, for IF), 53BP1 (NB100-305, for IF) from Novus Biologicals; TRF2 (sc271710, for IF), TIN2 (sc73177, for IF), TPP1 (sc100597, for IF and WB), RAP1 (sc53434, for WB), PPM1D (sc376257, for IF and WB), PPM1D (sc20712, for IF) from Santa Cruz Biotechnology; Phospho-Histone H2A.X (Ser139) (clone D7T2V, #80312), KAP1-S824 (#4127) and PPM1D (clone D4F7, 11901 for WB) from Cell Signaling Technology; γH2AX (05-636, for WB), GFP (11814460001, for WB), FLAG (F1804, for IF), Fk2 (04-263, for IF) from Roche. A custom-made pTRF2-S410 antibody was generated by immunization of rabbits with KLH-conjugated phospho-peptide RLVLEEDpSQSTEPSA corresponding to amino acids 403–417 of the human TRF2 (according to the numbering in reference sequence NP_005643.2) (Davids Biotechnologie). Subsequently, immune sera was affinity purified using negative and positive selection with non-phosphorylated and phosphorylated peptides, respectively. PPM1D inhibitor GSK2830371 was from MedChemExpress and was validated previously (44,49). Validated small molecule inhibitors of ATM (KU-55933), ATR (VE-821) and DNA-PK (NU7026) were from MedChemExpress and were used at final concentrations 10, 10 and 5 μM, respectively.

### Immunofluorescence microscopy

Cells grown on coverslips were washed in PBS, fixed by 4% PFA for 15 min and permeabilized with 0.2% Triton-X100 for 5 min. Where indicated, cells were pre-extracted prior fixation in 25 mM HEPES pH 7.4, 50 mM NaCl, 1 mM EDTA, MgCl_2_, 300 mM Sucrose, 0.5% Triton X-100 for 5 min. After washing in PBS, coverslips were blocked with 1% BSA in PBS for 30 min, incubated with primary antibodies for 2 h at room temperature and subsequently with Alexa Fluor secondary antibodies (Thermo Scientific) for 1 h. After incubation with DAPI for 2 min, coverslips were washed with water and mounted with Vectashield. For proximity ligation assay (PLA), coverslips were stained with the indicated primary antibodies followed by incubation with PLA probes (Merck, Duolink In Situ PLA Probe Anti-Rabbit PLUS and MINUS, DUO92002, DUO92004) for 1 h at 37 °C, ligation for 30 min at 37 °C, and polymerase reaction for 2 h at 37 °C according to the manufacturer's protocol (Merck, Duolink In Situ Detection Reagents Red, DUO92008). For immunofluorescence-FISH, coverslips were fixed, permeabilized, and blocked as described above. After dehydration with 70%, 95% and 100% ethanol for 3 min each, the coverslips were incubated for 10 min at 80°C face down on a slide with 20 μl of hybridization solution (10 mM Tris–HCl pH 7.2, 60% formamide, 0.4 μM TelC-Cy5 PNA probe (Panagene), and 0.5% blocking reagent (Roche, 10% stock in 100 mM maleic acid pH 7.5 and 150 mM NaCl). Hybridization was performed for 2 h at room temperature in a humidified chamber in dark. The coverslips were then washed twice for 10 min in wash buffer 1 (10 mM Tris–HCl pH 7.2, 70% formamide) and twice for 5 min in PBS. Incubation with primary antibodies was performed overnight at 4°C, followed with PBS wash and incubation with secondary antibodies for 1 h at room temperature. The coverslips were then stained with DAPI, rinsed in water and mounted using Vectashield. For the high content microscopy, images were acquired using Olympus ScanR equipped with 60×/1.42 OIL objective and analyzed using ScanR analysis software. Confocal imaging was performed using Leica DMi8 equipped with HC PL APO 63×/1.40 OIL CS2 objective. Images were acquired as Z-stacks of five planes with voxel size 44 × 44 × 129.7 nm and 3D-deconvolved using Huygens Professional (Scientific Volume Imaging) based on the theoretical point spread function. Metaphase spreads were imaged using Leica DM6000 equipped with a HCX PL APO 63×/1.40 OIL objective and a sCMOS Leica DFC 900 camera.

### Metaphase FISH

Cells were synchronised in late G2 phase by treatment with 9 μM R0-3306 (MedChemExpress) for 16 h. After washing with PBS, cells were released into media supplemented with 0.1 ug/ml colcemid (Sigma) and incubated for 3 h. Subsequently, cells were trypsinised, pelleted at 300 g for 5 min and resuspended in 5 ml of warm 75 mM KCl. After incubation for 30 min at 37°C, cell suspension was mixed with 1.25 ml of fixative solution (methanol:acetic acid, 3:1) while vortexing. After centrifugation, cells were 3× washed with fixative solution. Finally, cells were resuspended in 200–800 μl of fixative solution to achieve concentration 4 × 10^6^ cells/ml, and dropped onto frozen slides from distance of 30 cm. Slides were air dried overnight, washed 3 × 5 min in PBS and hybridisation was performed as described above. After washing in wash buffer 1 and three times 10 min in wash buffer 2 (100 mM Tris–HCl pH 7.2, 150 mM NaCl, 0.08% Tween 20), slides were stained with DAPI, PBS washed, dehydrated with 70%–95%–100% ethanol series, and mounted in Vectashield.

### Sample preparation for imaging of telomeric loops

For super-resolution imaging of telomeric loops, we used modified protocol from Doksani et al. Parental U2OS and PPM1D KO cells were trypsinized, washed with PBS and resuspended in 5 volumes of ice-cold nuclei extraction (NE) buffer (10 mM HEPES–KOH pH 7.9, 10 mM KCl, 1.5 MgCl_2_, 0.5 mM DTT, 0.5 mM PMSF) supplemented with cOmplete protease inhibitor cocktail (Roche). After 5 min of incubation, cells were pelleted at 500 g for 5 min at 4°C and resuspended in 2 volumes on NE buffer. Nuclei were released from cells using Dounce homogeniser and collected with centrifugation at 800 g for 5 min at 4°C. Nuclei were resuspended in nuclei wash (NW) buffer (10 mM Tris–HCl pH 7.4, 15 mM NaCl, 60 mM KCl, 5 mM EDTA) in concentration 1–2 × 10^7^ nuclei/ml, and incubated with 100 μg/ml of Trioxalen (Sigma) on ice while stirring in the dark for 5 min. Crosslinking was performed by exposing 2 ml of nuclei suspension at a 6-well plate to 365 nm light for 30 min on ice. Cross-linked nuclei were centrifuged at 800 g for 5 min at 4°C, washed with ice-cold NW buffer, and resuspended at 1 × 10^7^ nuclei/ml. The nuclei suspension was diluted 10× in spreading buffer (10 mM Tris–HCl pH 7.4, 10 mM EDTA, 0.05% SDS, 1 M NaCl) pre-warmed at 37°C, and 100 μl of the suspension was immediately dispersed on 13 mm round 1.5H coverslips using cytospin at 600 rpm for 2 min. Coverslips were dried at room temperature for 1 h and fixed in methanol:acetic acid (3:1) for 1h. Coverslips were dehydrated with 70%–95%–100% ethanol series and hybridized with TelC-Cy5 PNA probe overnight at 4°C in a humidified chamber protected from light. After washing with wash buffer 1 and wash buffer 2, coverslips were washed in water, air-dried and mounted with Vectashield.

### Structured illumination imaging

Three dimensional-structured illumination microscopy (3D-SIM) was performed using DeltaVision OMX™ V4 equipped with Blaze Module (GE Healthcare) and a PLAN APO N 60×/1.42 OIL objective. A 568 nm OPSL laser was used for excitation and a pco.edge 5.5 sCMOS camera for signal detection. Raw images were acquired in a z-stack with 125 nm step, 8 z slices, 15 images per slice, pixel size 80 nm. The image reconstruction was performed using SoftWorX software (GE Healthcare). Blinded analysis of telomeres in maximal projection images was done as previously described (33). Only telomere without gaps in telomere staining >500 nm were scored. Branched and overlapping telomeres (30–60% of molecules) were excluded from analysis.

### Proximity biotinylation assay and mass spectrometry

HEK293 stably transfected with empty pBIOID2 or pBIOID2-PPM1D-D314A were grown in media supplemented with 50 μM biotin for 5 h, then cells were collected, washed in cold PBS and lysed under denaturating conditions in lysis buffer (50 mM Tris pH 7.4, 1.0% SDS, 1 mM dithiothreitol, supplemented with cOmplete protease inhibitor). Protein lysates were diluted with four volumes of PBS and sonicated 3× for 30 s. Cell lysates were cleared by centrifugation at 15 000 g for 10 min and biotinylated proteins were pulled down by incubation with Dynabeads M-280 Streptavidin for 90 min. After washing twice in lysis buffer and twice with PBS, on-bead trypsin digestion was performed and peptides were analyzed by mass spectrometry using Orbitrap Fusion instrument (Q-OT- qIT, Thermo Scientific). All data were analyzed and quantified using MaxQuant (version 1.6.2.1) and Perseus softwares (50,51). Three biological replicates were analyzed and median peptide intensities were compared. Statistical significance was calculated using Student's t-test and hits with FDR <0.05 were considered significant.

### Immunoprecipitation

HEK293 cells were transfected with pEGFP, pEGFP-TRF2 or pEGFP-TRF1 and collected after 48 h. Cells were extracted by IP buffer (50 mM Tris pH 8.0, 150 mM NaCl, 1% Tween20, 0.1% NP-40, 10% glycerol, 2 mM EDTA, 3 mM EGTA, 10 mM MgCl_2_) supplemented with PhosSTOP and protease inhibitors (Roche), sonicated and DNA was digested by Bensonase. Cell extracts were incubated with GFP Trap beads (Chromotek) for 1 h and after washing, proteins were eluted by Laemli buffer and analyzed by immunoblotting.

### *In vitro* phosphatase assay

Expression and purification of human His-PPM1D was described previously (52). EGFP-TRF2 was purified from transiently transfected U2OS cells using GFP trap and a high salt IP buffer (50 mM Tris pH 8.0, 1 M NaCl, 1% Tween20, 0.1% NP-40, 10% glycerol, 2 mM EDTA, 3 mM EGTA, 10 mM MgCl_2_) supplemented with PhosSTOP and protease inhibitors. Beads were washed with a phosphatase buffer and incubated with mock or with 150 ng of the purified His-PPM1D for 20 min at 37°C. Reaction was stopped by addition of 4× concentrated Laemli buffer.

### Peptide pull down

Biotin-Ahx-ISRLVLEEDpSQSTEPSAGLN-amide (TRF2-pS410) and Biotin-Ahx-ISRLVLEEDSQSTEPSAGLN-amide (TRF2-CTRL) peptides were synthesized (Genscript), dissolved in ammonia water and then diluted to 1 mg/ml in TBST (150 mM NaCl, 3 mM KCl, 25 mM Tris pH 8.0, 10% glycerol, 1 mM DTT, 0.1% Tween20). Peptide pull down was performed as described (53). Dynabeads M-280 Streptavidin (Thermo Fisher Scientific) were incubated with peptides (20 μg) in TBST for 60 min and then beads were washed 3 times with TBST. Coupled beads were incubated with Hela nuclear extract (6 mg/ml, IpraCell) for 90 min at 4°C and then were washed 3 times with TBST and once with PBS. Proteins bound to the beads were digested by trypsin and peptides were analyzed by mass spectrometry. Three independent experiments were compared in one MS measurement.

### Fluorescence anisotropy assay

Purification of human TIN2 was described previously (54). TRF2-pS410 and TRF2-CTRL peptides fluorescently labelled at N-terminus with carboxyfluorescein (FAM; λ_ex_ 494 nm, λ_em_ 518 nm) were synthesized by GenScript. Peptides (3 nM) in a 1.5 ml quartz-glass cuvette with a magnetic stirrer were titrated with TIN2 to a final concentration of 500 nM in 50 mM NaCl in 50 mM phosphate buffer, pH 7.0 at 25°C. Fluorescence anisotropy change upon addition of TIN2 was measured at λ_ex_ 490 nm, λ_em_ 520 nm with excitation and emission slits 9 nm. Fluorescence anisotropy was measured three times, and averaged with a relative standard deviation always lower than 3%. The value of the dissociation constant was determined by non-linear least square fits according to the equation: *r* = *r*^MAX^*c* / (*K*_D_ + *c*) using OriginPro 2022 (OriginLab Corporation) (20).

### Cell proliferation assay

Cell survival assay was performed as described (10). Briefly, cells were seeded to 96-well plates at 100–130 cells/well, and treated as indicated. Seven days after treatment, resazurin was added in fresh media at final concentration 30 μg/ml. Fluorescence at excitation wavelength 560 nm and emission 590 nm was measured using Envision plate reader (PerkinElmer, Waltham, MA, USA) after 2 h incubation.

### Statistical analysis

Statistic was calculated using PRISM 5 (GraphPad Software). Only two-tailed test were used. Student's t-test were performed under the assumption of normality. As a nonparametric test, we used Mann–Whitney statistics. All experiments were reproduced with similar results at least two times.

## RESULTS

### PPM1D interacts with components of the shelterin complex

**Figure 1.**
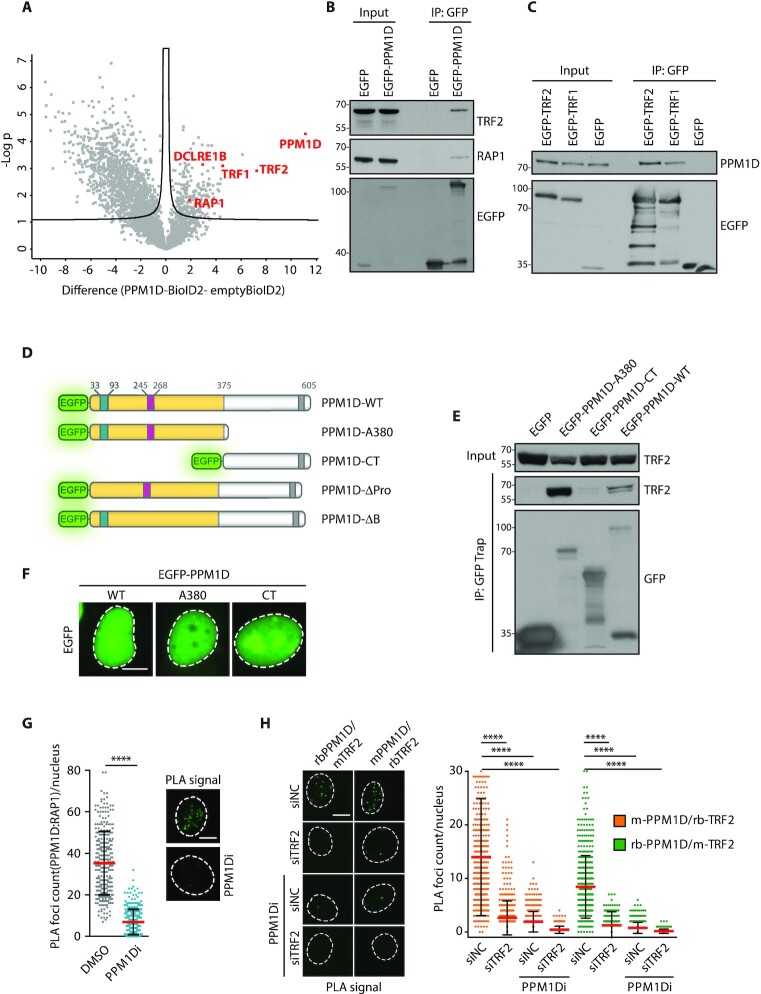
PPM1D interacts with component of the shelterin complex. (**A**) HEK293 cells stably expressing PPM1D-D314A-BioID2 or empty BioID2 were lysed 5h after treatment with biotin. Biotinylated proteins were pulled down by streptavidin beads and bound proteins were analyzed by MS (*n* = 3). Volcano plot shows –log *P* values for proteins enriched or reduced in PPM1D-BioID2 sample. Line delineates the statistical significance (FDR < 0.05). (**B**) HEK293 cells were lysed 24 h after transfection with plasmids expressing EGFP or EGFP-PPM1D and cell extracts supplemented with bensonase were incubated with GFP trap. Bound proteins were analyzed by immunoblotting. (**C**) MCF7 cells were transfected with plasmids expressing EGFP, EGFP-TRF1, or EGFP-TRF2. Cell extracts supplemented with bensonase were incubated with GFP trap. Binding of PPM1D was probed by immunoblotting. (**D**) Scheme of EGFP-tagged PPM1D constructs used in the study. Shown are the catalytic domain in yellow, the basic loop in magenta, the Proline-rich loop in cyan and the NLS in grey. Note that an additional NLS is located within the B loop. (**E**) HEK293 cells were transfected with plasmids expressing EGFP, the wild type EGFP-PPM1D, EGFP-PPM1D-A380 corresponding to the catalytic domain, or EGFP-PPM1D-CT corresponding to the unstructured C-terminal tail of PPM1D. Cell extracts were incubated with GFP trap and binding of TRF2 was evaluated by immunoblotting. (**F**) U2OS were transfected with plasmids coding for EGFP-PPM1D variants. Cells were fixed and visualized by wide-field microscopy, the scale bar represents 10 μm. Representative images are shown. (**G**) MCF7 cells were fixed and probed for interaction of PPM1D with RAP1 by PLA assay. Where indicated, cells were treated with PPM1D inhibitor for 24 h. Mean count on nuclear PLA foci is plotted ± SD, *n* = 300. Statistical significance was evaluated using Mann–Whitney test, (****P < 0.0001). Representative experiment is shown from two independent repeats. The scale bar in representative images corresponds to 10 μm. (**H**) MCF7 cells were transfected twice with control siRNA (siNC) or siRNA to TRF2. After 6 days, cells were fixed and probed for interaction of PPM1D with TRF2 by PLA assay using two different pairs of antibodies (rabbit rb-PPM1D/mouse m-TRF2, mouse m-PPM1D/rabbit rb-TRF2). Where indicated, cells were treated with PPM1D inhibitor for 18 h prior fixation. Mean count of the nuclear PLA foci is plotted ± SD, *n* = 500. Statistical significance was evaluated using Mann–Whitney test (****P < 0.0001). Representative experiment is shown from two independent repeats. The scale bar in representative images corresponds to 10 μm.

Protein phosphatase magnesium-dependent 1 delta (PPM1D) is a chromatin-bound protein with poor solubility making analysis of its interacting partners a major challenge (8). To identify proteins forming a complex with PPM1D, we established a stable HEK293 cell line expressing a phosphatase-dead PPM1D-D314A fused with a proximity-dependent biotin identification (BioID2) tag and control cells expressing empty BioID2 (55,56). After incubating with biotin, cells were extracted under denaturating conditions and biotinylated proteins were isolated using streptavidin beads and subsequently identified by mass spectrometry (Figure 1A, Supplementary Table S1). This analysis revealed that three components of the shelterin complex (namely TRF2, TRF1 and RAP1) and telomere-associated exonuclease DCLRE1B (also known as Apollo) were significantly enriched in complex with PPM1D-D314A-BioID2 fusion protein. To confirm the results from the proximity biotin labelling assay, we performed immunoprecipitation from HEK293 cells expressing EGFP-PPM1D or empty EGFP. We found that EGFP-PPM1D specifically interacted with TRF2 and RAP1 (Figure 1B). In addition, EGFP-TRF2 and EGFP-TRF1 pulled down endogenous PPM1D from MCF7 cells indicating that PPM1D interacts with shelterin in various cell types (Figure 1C). To map the interaction between PPM1D and the shelterin, we performed immunoprecipitation with the full length EGFP-PPM1D, a mutant containing the catalytic domain of PPM1D (PPM1D-A380) or a mutant comprising of the unstructured C-terminal region of PPM1D (PPM1D-CT) (Figure 1D, E). Due to the presence of two NLS sequences located at the C-terminal region and within the B-loop, respectively, the catalytic domain of PPM1D as well as the C-terminal fragment of PPM1D localized in the nucleus (Figure 1F) (57). However, only the catalytic domain of PPM1D but not the C-terminal tail co-immunoprecipitated with TRF2 (Figure 1E). Moreover, isolated EGFP-TRF2 (but not EGFP alone) was able to pull down purified His-PPM1D *in vitro*, suggesting that the interaction between TRF2 and PPM1D is direct (Supplementary Figure S1A).

Finally, we tested the interaction between PPM1D and shelterin in cells using a proximity ligation assay (43). We observed distinct nuclear foci in MCF7 cells when probing for PPM1D and RAP1 (Figure 1G). Similarly, two distinct sets of antibodies directed against PPM1D and TRF2 showed a strong nuclear PLA signal in MCF7 and U2OS cells (Figure 1H, Supplementary Figure S1B). Importantly, the specificity of the observed PLA signal was confirmed by a strong reduction caused by treating cells with GSK2830371 (further referred to as PPM1Di) that promotes a proteasomal degradation of PPM1D (Figure 1G, Supplementary Figure S1C) (44,49). Similarly, depletion of TRF2 by RNA interference suppressed the PLA signal thus supporting our conclusion that PPM1D and TRF2 interact in the cell nuclei (Figure 1H).

Taken together, we conclude that PPM1D interacts through its catalytic domain with several components of the shelterin complex in various cell types regardless of the type of telomere maintenance, including telomerase proficient MCF7 cells and alternative telomere lengthening (ALT)-dependent U2OS cells.

### PPM1D is present at telomeres

**Figure 2.**
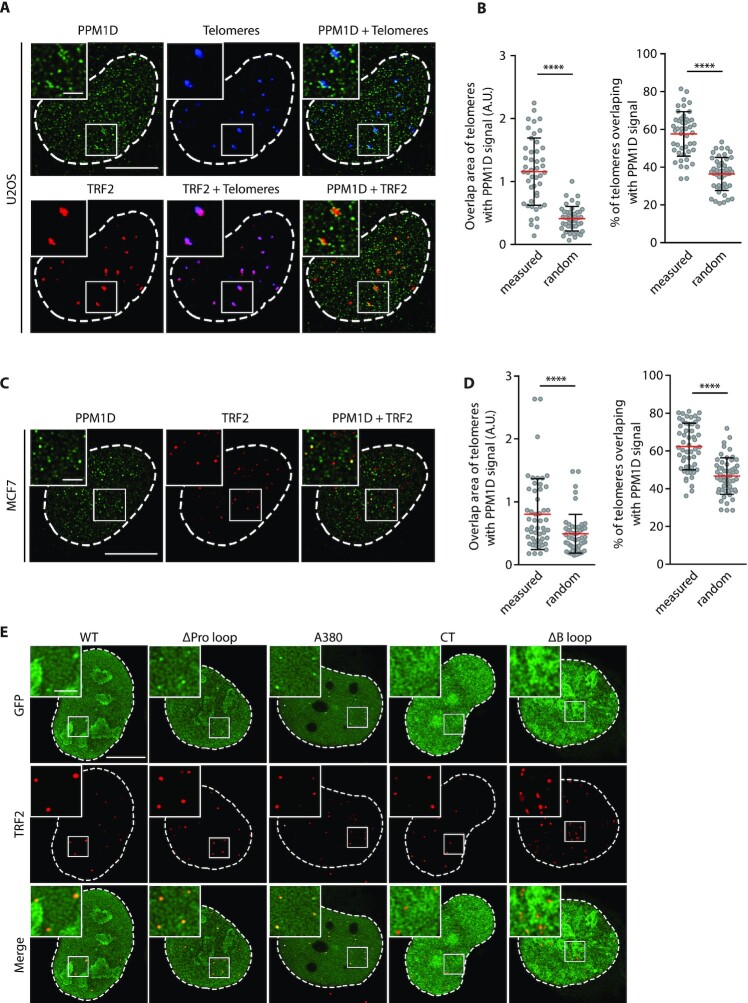
PPM1D is present at telomeres. (**A**) U2OS cells were co-transfected with plasmids coding for mCherry-dCas9 and telomeric repeat-targeting sgRNA. After 24h, cells were fixed and stained for PPM1D and TRF2. Images show a single confocal plane processed with deconvolution. The scale bars represent 10 μm or 2 μm, respectively. (**B**) Quantification of A. Area of the overlapping PPM1D and TRF2 signal was determined using Interaction Factor package in ImageJ. Subsequently, PPM1D signal was randomized for each image. Means of 20 randomizations are plotted together with experimentally observed values (left). Shown is also a fraction of telomeres that contain PPM1D signal (right). Values for 46 cells form two independent experiments are plotted with means ± SD. Statistical significance was evaluated using paired *t*-test (****P < 0.0001). (**C**) MCF7 cells were stained for PPM1D and TRF2 and imaged by confocal microscopy. A representative single deconvolved planes are shown. The scale bar represents 10 μm or 2 μm respectively. (**D**) PPM1D and TRF2 signals from (C) were analyzed as in (B). Values for 51 cells form two independent experiments are plotted with means ± SD. Statistical significance was evaluated using paired *t*-test (****P < 0.0001). (**E**) U2OS cells were transfected with plasmids coding for individual EGFP-PPM1D variants. Cells were fixed, stained for TRF2 and imaged by confocal microscopy. A representative single deconvolved planes are shown. The scale bar represents 10 or 2 μm, respectively.

Apart from functions at telomeres, TRF2 and TRF1 were reported to localize also to other chromatin compartments (58–61). Therefore, we wondered where the interaction between PPM1D and the shelterin complex occurred at subcellular level. To this end, we transfected U2OS cells with a plasmid expressing an enzymatically inactive dCas9-mCherry reporter together with a telomere-specific sgRNA and we visualized telomeres by confocal microscopy (62). In parallel, we probed cells with validated antibodies against PPM1D and TRF2 (Supplementary Figure S1D, E) (8). As expected, signal from the dCas9-mCherry telomeric reporter overlapped with the staining for TRF2 (Figure 2A). As expected, we observed a dotted nuclear pattern reflecting the localization of PPM1D to the chromatin (Figure 2A) (8). In addition, we noticed that a fraction of spots recognized by PPM1D antibody localized at telomeres (Figure 2A). To investigate possible contribution of the stochastic cluster overlap, we randomized PPM1D signal distribution using Interaction Factor package in ImageJ and compared random overlap with non-random values (63). We confirmed that the experimentally observed overlap between PPM1D and the telomeric staining in U2OS cells was statistically significant (Figure 2B). In addition, we observed that PPM1D was present at approximately 60% of telomeres probably reflecting a dynamic interaction between PPM1D and the shelterin complex (Figure 2B). Finally, we used an identical experimental approach to determine PPM1D distribution in MCF7 cells (Figure 2C). We noted that TRF2 foci in MCF7 cells were smaller than in U2OS cells probably reflecting shorter telomeres in MCF7 cells compared to the ALT-positive U2OS cells. Nevertheless, we found that a fraction of endogenous PPM1D localized at telomeres recognized by TRF2 staining in MCF7 cells (Figure 2D). Interestingly, the fraction of telomeres positive for PPM1D was comparable in MCF7 and U2OS cells (Figure 2D). In summary, we conclude that PPM1D can associate with telomeres in various cell types.

To identify the regions in PPM1D that are necessary for its localization at telomeres, we transfected cells with plasmids expressing EGFP-tagged PPM1D or its deletion mutants. We found that the wild-type EGFP-PPM1D, a deletion mutant lacking the Proline-rich region (referred to as ΔPro) and a PPM1D-A380 mutant comprising of the catalytic domain between amino acids 1–380 all were enriched in TRF2 foci (Figure 2E, Supplementary Figure S1F, G). In contrast, the unstructured C-terminal fragment of PPM1D failed to accumulate in TRF2-positive foci although it showed a strong nuclear staining (Figure 2E). Finally, a deletion mutant lacking amino acids 246–251 of the B loop (referred to as ΔB) localized to the nucleus but it failed to co-localize with TRF2 (Figure 2E, Supplementary Figure S1F, G). Thus, the microscopic analysis revealed that the B loop in the catalytic domain of PPM1D mediates its localization at telomeres, which is in good agreement with the data from immunoprecipitation (Figure 1E). In addition, the observed difference between intensities of the wild-type EGFP-PPM1D and the EGFP-PPM1D-A380 mutant suggests that the C-terminal tail of PPM1D may be involved in negative regulation of PPM1D localization at telomeres.

### PPM1D counteracts ATR-dependent phosphorylation of TRF2 at S410

**Figure 3.**
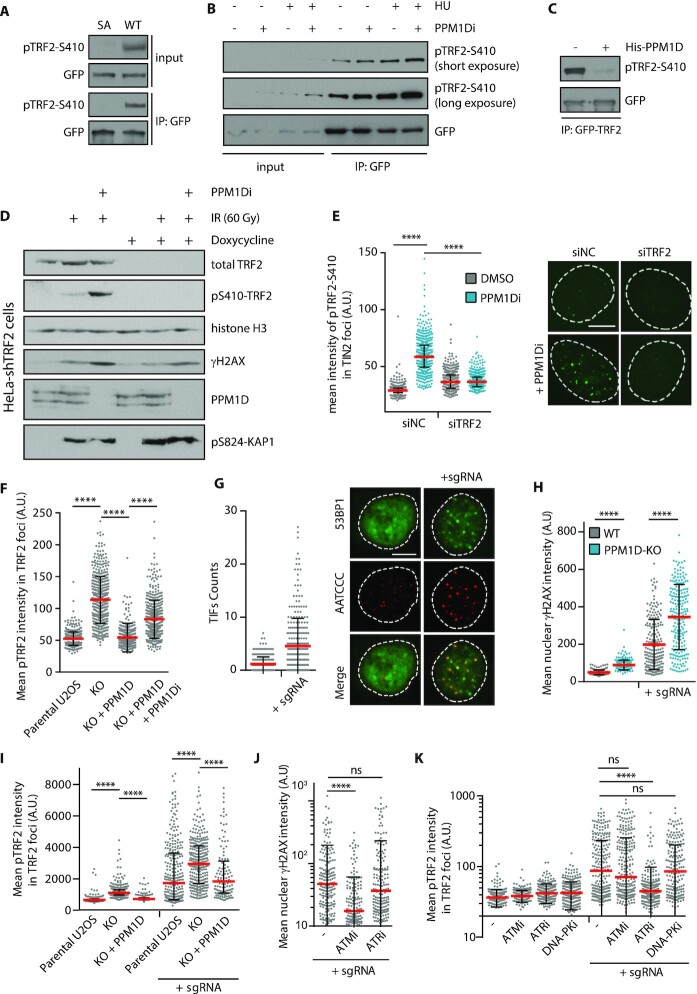
TRF2 is phosphorylated at S410 by ATR and dephosphorylated by PPM1D. (**A**) HEK293 cells were transfected with the wild-type EGFP-TRF2 (WT) or EGFP-TRF2-S410A (SA) mutant and incubated with PPM1Di for 18 h prior harvesting. Cell extracts were incubated with GFP trap and bound proteins were analyzed by immunoblotting. (**B**) HEK293 stably expressing EGFP-TRF2 were treated with DMSO, HU (2 mM), PPM1Di (1 μM) or combination of both for 18 h. Cell extracts were incubated with GFP trap and bound proteins were analyzed by immunoblotting. (**C**) *In vitro* phosphatase assay. EGFP-TRF2 was isolated from cells by GFP Trap in a buffer containing 1 M NaCl. Beads were washed with a phosphatase buffer and incubated with mock or with the purified His-PPM1D for 20 min at 37°C. Level of TRF2-S410 phosphorylation was analyzed by immunoblotting. (**D**) HeLa cells stably transfected with inducible TRF2 shRNA were treated with mock or with doxycycline (2 μg/ml) for 7 days and were exposed or not to IR (60 Gy). Where indicated, cells were incubated with PPM1Di for the last 12 h. Nuclear extracts were separated on 4–20% SDS-PAGE gel and analyzed by immunoblotting. (**E**) U2OS cells after two consecutive transfections of control or TRF2 siRNA were treated or not with PPM1D inhibitor (2 μM, 4 h), fixed and co-stained for TIN2 (telomeric marker) and pTRF2-S410. Plotted is the mean pTRF2-S410 intensity in TIN2-positive foci, each dot represents a single cell (n = 500). Bars indicate mean ± SD, statistical significance was evaluated using Mann–Whitney test (****P < 0.0001). Representative experiment is shown from two independent repeats. The scale bar in representative images corresponds to 10 μm. (**F**) Parental U2OS, U2OS-PPM1D-KO and U2OS-PPM1D-KO cells stably expressing FLAG-PPM1D were treated or not with PPM1D inhibitor for 24 h. Cells were pre-extracted, fixed and stained for TRF2 and pTRF2-S410. Plotted is the mean pTRF2-S410 intensity in TRF2-positive foci, each dot represents a single cell (*n* = 300). Bar indicates mean ± SD, statistical significance was evaluated using Mann–Whitney test (****P < 0.0001). Representative experiment is shown from two independent repeats. (**G**) U2OS cells were transfected with plasmids coding for Cas9-EGFP with or without the telomeric repeat-targeting sgRNA. After 24 h, cells were fixed, hybridized with telomeric FISH probe, and stained for 53BP1 (TIF marker). The scale bar represent 10 μm). Bar indicates mean ± SD, *n* = 300. (**H**) Parental U2OS or U2OS-PPM1D-KO cells were transfected with plasmids coding for Cas9-EGFP with or without telomeric repeat-targeting sgRNA. After 24 h, cells were fixed and stained for γH2AX. Mean nuclear intensity is plotted ± SD, *n* ≥ 208. Statistical significance was evaluated using Mann–Whitney test (****P < 0.0001). Representative experiment is shown from three independent repeats. (**I**) U2OS cells were transfected as in H and were stained for TRF2 and pTRF2-S410. Plotted is the mean pTRF2-S410 intensity in TRF2-positive foci. Bars indicate mean ± SD, *n* ≥ 150. Statistical significance was evaluated using Mann–Whitney test (****P < 0.0001). Representative experiment is shown from two independent repeats. (**J**) U2OS cells were transfected with plasmids coding for Cas9-EGFP with or without the telomeric repeat-targeting sgRNA and were treated with indicated inhibitors for 20 h. After fixation, the intensity of γH2AX signal in TRF2 foci was determined by ScanR microscopy. Bars indicate median ± SD, *n* ≥ 161. Statistical significance was evaluated using Mann–Whitney test (****P < 0.0001). Representative experiment is shown from two independent repeats. (**K**) U2OS cells were treated as in (J) and were probed with TRF2 and pTRF2-S410 antibodies. Plotted is the mean pTRF2-S410 intensity in TRF2 foci. Bars indicate median ± SD, *n* ≥ 249. Statistical significance was evaluated using Mann–Whitney test (****P < 0.0001). Representative experiment is shown from two independent repeats.

As PPM1D localizes at telomeres, we wondered if it could regulate the phosphorylation of the shelterin components either in context of the cell cycle progression or following DNA damage at telomeres. Since PPM1D has been implicated in termination of the global DNA damage response, we have focused on the phosphorylations triggered by exposure of cells to genotoxic stress. Unfortunately, commercial antibodies raised against several phosphopeptides in TRF2 and TRF1 did not show sufficient level of sensitivity and specificity preventing us from testing the effect of PPM1D activity (data not shown). Therefore, we generated an affinity purified rabbit polyclonal antibody against the phosphorylated S410 of TRF2 that is conserved across species, matches a consensus motif for ATM/ATR and PPM1D and has previously been detected in cells exposed to ionizing radiation or to treatment with cytarabine (Supplementary Figure S1H) (64–66). First, we tested the reactivity of pTRF2-S410 antibody using the wild-type EGFP-TRF2 or the EGFP-TRF2-S410A mutant immunopurified from HEK293 cells. Importantly, we observed a strong reduction of the signal in the alanine mutant, confirming that the pTRF2-S410 antibody predominantly recognizes the phosphorylated form of TRF2 in immunoblotting (Figure 3A). In addition, we found that the basal level of pTRF2-S410 signal that was increased by treatment of the cells with hydroxyurea and/or PPM1D inhibitor which is consistent with the DNA damage-induced phosphorylation of TRF2 that is counteracted by PPM1D (Figure 3B). In agreement with this possibility, we found that purified His-PPM1D dephosphorylated the purified TRF2 at S410 *in vitro* (Figure 3C). Next, we used control HeLa cells or cells with doxycycline-induced knock-down of TRF2 and exposed them to ionizing radiation (60 Gy) (45). In non-treated cells, the phosphorylation of endogenous TRF2 was below the detection limit in the nuclear extracts. On the other hand, the extensive DNA damage induced the signal of pTRF2-S410 antibody and importantly, the specificity was confirmed by depletion of the TRF2 (Figure 3D). As expected, treatment of cells with PPM1Di decreased the level of PPM1D protein and induced γH2AX staining (8,44,49). In addition, we found that inhibition of PPM1D further increased the pTRF2-S410 signal suggesting that PPM1D might dephosphorylate pTRF2-S410 (Figure 3D). To validate specificity of the pTRF2-S410 antibody in immunofluorescence microscopy, we depleted endogenous TRF2 in U2OS cells by RNAi and treated them or not with PPM1D inhibitor (Figure 3E). As expected, we observed a strong induction of the pTRF2-S410 signal at telomeres upon treatment of control cells with PPM1D inhibitor. Importantly, the signal was lost upon depletion of TRF2, thus confirming specificity of the antibody (Figure 3E). Further, we observed an increase in pTRF2-S410 phosphorylation in U2OS-PPM1D-KO cells and the signal was significantly reduced by expression of the FLAG-PPM1D confirming that the observed phenotype was indeed caused by the loss of PPM1D (Figure 3F). We conclude that PPM1D counteracts the TRF2-S410 phosphorylation at telomeres.

As the basal level of pTRF2-S410 signal in non-treated cells was relatively low, we searched for conditions that would stimulate the phosphorylation of TRF2. Upon exposure to ionizing radiation, DSBs are randomly generated across the genome making interpretation of events observed at telomeres difficult. To induce DSBs specifically at telomeres, we transfected cells with a plasmid expressing Cas9 and a sgRNA targeting the telomeric repeats (67). Consistent with previous reports, we observed formation of the telomeric dysfunction-induced foci (TIFs) defined by recruitment of 53BP1 and by phosphorylation of H2AX at S139 (called γH2AX) (Figure 3G, H, Supplementary Figure S1I) (68). As expected, DSBs induction eventually resulted in telomere clustering that we observed as reduced telomere count and increased area of the foci (Supplementary Figure S2A–C) (69). In addition, we noted an increased γH2AX signal in cells lacking PPM1D, which is in agreement with the previously described role of PPM1D in dephosphorylating H2AX at chromatin (Figure 3H, Supplementary Figure S1I) (8). Importantly, telomeric DNA damage also strongly induced the TRF2-S410 phosphorylation at telomeres and the signal was further increased in U2OS-PPM1D-KO cells (Figure 3I, Supplementary Figure S2D). Of note, pTRF2-S410 signal was significantly enriched at telomeres in U2OS-PPM1D-KO cells without any induction of telomeric damage suggesting that PPM1D may constantly dephosphorylate TRF2 at telomeres (Figure 3I, Supplementary Figure S2D).

Finally, we induced DSBs at telomeres in cells treated with small molecule inhibitors of the major protein kinases responding to DNA damage and assayed the impact on protein phosphorylation at telomeres. Similarly to DSBs induced by TRF1-FokI, we observed that inhibition of ATM reduced the level of γH2AX at telomere breaks induced by Cas9 (Figure 3J) (70). In contrast, pTRF2-S410 phosphorylation was insensitive to the inhibition of ATM but was reduced upon treatment with ATR inhibitor (Figure 3K, Supplementary Figure S2E). Similarly, we observed that RNAi-mediated depletion of ATR (but not ATM) supressed the level of pTRF2-S410 phosphorylation (Supplementary Figure S2F–H). We conclude that following induction of DSBs at telomeres, TRF2 phosphorylation at S410 is inversely regulated by ATR and PPM1D.

### TRF2 phosphorylation at S410 increases its binding to TIN2

**Figure 4.**
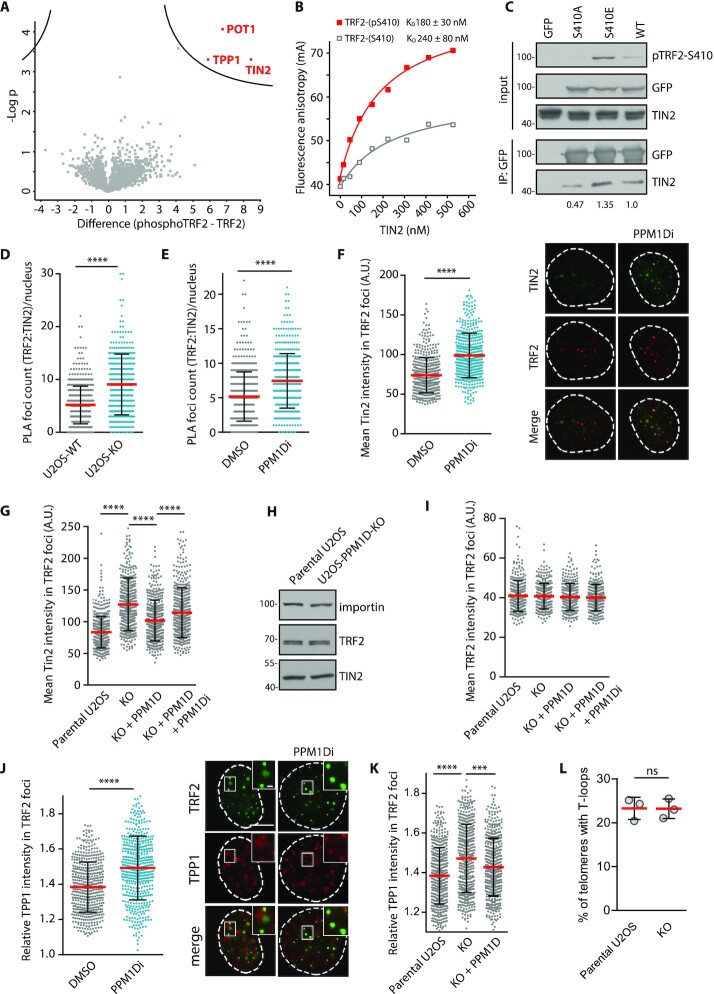
TRF2 phosphorylation at S410 increases its binding to TIN2. (**A**) Biotin-Ahx-ISRLVLEEDpSQSTEPSAGLN (TRF2-pS410) and Biotin-Ahx–SRLVLEEDSQSTEPSAGLN (TRF-CTRL) peptides were incubated with nuclear extracts and pulled down by streptavidin beads. Bound proteins were identified by mass spectrometry (*n* = 3). Plotted are –log *P* values of proteins enriched or reduced in condition with TRF2-pS410 peptide. The line delineates the statistical significance (FDR < 0.1). (**B**) Fluorescently-labelled TRF2-pS410 and TRF2-CTRL peptides were titrated with purified TIN2 to a final concentration of 500 nM. Fluorescence anisotropy change was measured and dissociation constant values for unmodified and modified oligopeptides were calculated as described in Methods. (**C**) HEK293 cells stably expressing EGFP-TRF2 were treated with DMSO or with PPM1D inhibitor for 4 h. EGFP-TRF2 was immunoprecipitated from cell extracts with GFP Trap. Proteins were separated by SDS-PAGE and binding of TIN2 was determined by immunoblotting. Numbers at the bottom indicate the TIN2 signal relative to the total immunoprecipitated TRF2 and normalized to the wild-type TRF2. Representative result from three experiments is shown. (**D**) TRF2:TIN2 interaction was determined in parental U2OS and U2OS-PPM1D-KO cells by PLA. Mean PLA foci count is plotted ± SD, *n* = 500. Statistical significance was evaluated using Mann–Whitney test (****P < 0.0001). Representative experiment is shown from two independent repeats. (**E**) TRF2:TIN2 interaction was determined in U2OS cells treated with DMSO or PPM1Di by PLA. Mean PLA foci count is plotted ± SD, *n* = 500. Statistical significance was evaluated using Mann–Whitney test (****P < 0.0001). Representative experiment is shown from two independent repeats. (**F**) U2OS cells were treated or not with PPM1Di for 24 h, pre-extracted, fixed and stained with TRF2 (m-Santa Cruz) and TIN2 (Rb-Novus) antibodies. Mean TIN2 intensity in TRF2 foci is plotted ± SD, *n* = 300. Statistical significance was evaluated using Mann–Whitney test (****P < 0.0001). Representative experiment is shown from two independent repeats. The scale bar represents 10 μm. (**G**) Parental U2OS, U2OS-PPM1D-KO and U2OS-PPM1D-KO stably expressing FLAG-PPM1D cells were treated or not with PPM1Di for 24 h. Cells were pre-extracted, fixed and stained for TIN2 and TRF2. Mean TIN2 intensity in TRF2 foci ± SD is plotted, *n* = 300. Statistical significance was evaluated using Mann–Whitney test (****P < 0.0001). Representative experiment is shown from two independent repeats. (**H**) Levels of TRF2 and TIN2 were analyzed in whole cell extracts from the parental U2OS and U2OS-PPM1D-KO cells by immunoblotting. Importin staining was used as a loading control. (**I**) Cells from G were analysed for TRF2 intensity in TRF2 foci. Plotted is mean ± SD, *n* = 300. (**J**) U2OS cells were treated or not with PPM1Di for 24 h, pre-extracted, fixed and stained with TRF2 and TPP1 antibodies. Mean TPP1 intensity in TRF2 foci normalized to the mean nuclear TPP1 intensity ± SD is plotted, *n* > 300. Statistical significance was evaluated using Mann–Whitney test (****P < 0.0001). The scale bars in representative images corresponds to 10 μm and 1 μm respectively. (**K**) Parental U2OS, U2OS-PPM1D-KO cells and U2OS-PPM1D-KO stably expressing FLAG-PPM1D cells were pre-extracted, fixed and stained for TPP1 and TRF2. Mean TPP1 intensity in TRF2 foci normalized to the mean nuclear TPP1 intensity ± SD is plotted, *n* > 300. Statistical significance was evaluated using Mann–Whitney test (****P < 0.0001, ***P < 0.001). (**L**) Chromosome spreads from parental U2OS and U2OS-PPM1D-KO cells were hybridized with TAACCC FISH-probe and imaged by 3D-SIM. Plotted is a fraction of telomeres that formed t-loops. More than 203 telomeres were quantified per condition in each experiment (*n* = 3). Significance was determined by unpaired *t*-test.

Several recent studies have identified regions within individual shelterin components that mediate protein–protein interactions and are critically needed for folding of the shelterin complex (16,25,30,31,71). For instance, the TRF1_TRFH_ (residues 58–268) and TRF2 _TRFH_ (residues 84–287) domains interact with a TRFH-binding motif (TBM) of TIN2 (residues 256–276) (30). In addition, TRFH domain of TIN2 interacts with a recently described TBM2 region of TRF2 (residues 392–408) (31). As the published crystal structure of TIN2_TRFH_-TRF2_TBM2_ interaction interface lacks the structural information for S410, we used Alphafold2 Colab to predict the position of residues 392–420 of TRF2 (30). The structural alignment of Alphafold2 model showed a perfect overlap with the crystal structure (with RMSD 0.252 Å) (Supplementary Figure S3A). In this model, S410 of TRF2 is positioned opposite the positively charged residues of TIN2_TRFH_ suggesting that phosphorylation of S410 might strengthen the TRF2-TIN2 interaction by formation of salt bridges between the phosphate and basic residues in the AA50–56 region of TIN2 (Supplementary Figure S3A). To experimentally test the impact of TRF2_TBM2_ phosphorylation on TRF2-TIN2 interaction, we designed several independent assays. First, we performed a pull down from the nuclear extracts using biotinylated phosphorylated or non-phosphorylated peptides of TRF2 as baits. Mass spectrometry analysis revealed that the phosphorylated but not the non-phosphorylated TRF2 peptide specifically pulled down TIN2, TPP1 and POT1 (Figure 4A, Supplementary Table S2). Second, we quantified the binding affinities of the purified TIN2 with short, fluorescently labelled TRF2 oligopeptides containing phosphorylated or non-phosphorylated S410 (Figure 4B). Analysis of the binding isotherms showed that the binding affinity for unmodified TRF2-S410 oligopeptide was *K*_D_ = 240 ± 80 nM that corresponded well to the previously reported affinity for TRF2-TIN2 binding (71). When S410 was phosphorylated, we observed a significant increase of the binding affinity with the corresponding *K*_D_ = 180 ± 30 nM. To confirm the data from the *in vitro* assays, we tested the interaction between TRF2 and TIN2 in cells by immunoprecipitation (Figure 4C). Consistent with the previous reports, we observed that the wild-type EGFP-TRF2 interacted with TIN2 (25). In addition, the non-phosphorylatable EGFP-TRF2-S410A mutant pulled down a reduced but still detectable level of TIN2, suggesting that modification of S410 is not absolutely required for the basal interaction between TRF2-TIN2 (Figure 4C). This finding is in agreement with the previous report where interaction was observed with a TRF2_TBM2_ fragment (residues 392–408) lacking the S410 site (72). Interestingly, however, we observed an increased interaction between the phosphorylation mimicking EGFP-TRF2-S410E mutant, which is consistent with increased binding affinity between TRF2 and TIN2 upon phosphorylation (Figure 4C).

To test if the TRF2 interaction with TIN2 is regulated by PPM1D, we performed the PLA assay in parental U2OS cells, U2OS-PPM1D-KO and U2OS cells treated with PPM1D inhibitor. We observed that loss or inhibition of PPM1D significantly increased the interaction between TRF2 and TIN2 (Figure 4D, E, Supplementary Figure S3B). Consistent with this, we found that TIN2 was enriched at telomeres in U2OS cells treated with PPM1D inhibitor compared to the non-treated cells (Figure 4F). Similarly, intensity of the TIN2 signal at telomeres was increased in U2OS-PPM1D-KO cells compared to the parental U2OS cells and the level was rescued by expression of the wild-type EGFP-PPM1D (Figure 4G). Importantly, total protein levels of TRF2 and TIN2 remained unchanged in U2OS-PPM1D-KO cells thus excluding the possibility that the observed enrichment of TIN2 at telomeres is a consequence of altered protein expression (Figure 4H). In contrast to TIN2, we did not observe any increase in TRF2 accumulation at telomeres in cells lacking PPM1D suggesting that the increased recruitment of TIN2 depends on phosphorylation status of TRF2 rather than a change of its total levels at the telomere (Figure 4I). As TIN2 mediates the recruitment of TPP1-POT1 to TRF1/2 we also evaluated the amount of TPP1 at telomeres. We observed that inhibition or loss of PPM1D increased the level of TPP1 at telomeres confirming that the activity of PPM1D may regulate assembly of the shelterin complex at telomeres (Figure 4J, K). Similarly to U2OS cells, we observed that inhibition of PPM1D increased the phosphorylation of TRF2-S410 as well as the levels of TIN2 and TPP1 at telomeres in MCF7 cells, suggesting that the impact of PPM1D activity on recruitment of shelterin components to telomeres is not restricted to cells with alternative lengthening of telomeres (Supplementary Figure S3C, D and E).

TRF2 and TIN2 jointly protect telomeric ends by promoting formation of t-loop and therefore we asked if manipulation with the strength of TRF2:TIN2 binding by removing PPM1D activity could affect t-loop formation. To this end, we prepared chromatin spreads from the parental U2OS and U2OS-PPM1D-KO cells and determined fractions of the linear or looped chromosome ends by 3D-SIM microscopy as previously described (33). Consistent with published literature, we observed t-loops in about 25% of chromosomes (33,73). However, we did not find any significant differences between parental U2OS and U2OS-PPM1D-KO cells (Figure 4L, Supplementary Figure S3F) suggesting that PPM1D activity does not interfere with formation of the t-loop. On the other hand, we cannot exclude that differences in organisation of the chromosome ends caused by loss of PPM1D are below the sensitivity of the assay because we were unable to conclusively categorize about a half of the imaged telomeres.

### Increased PPM1D activity impairs assembly of the shelterin complex

**Figure 5.**
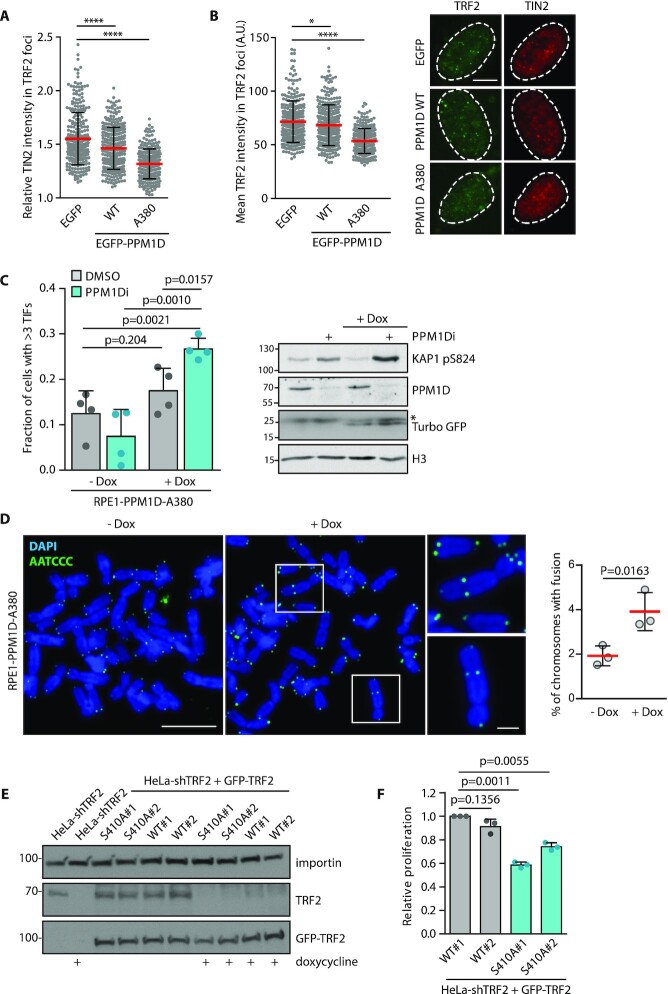
Increased PPM1D activity at telomere impairs shelterin function. (**A**) U2OS cells were fixed 24 h after transfection with the wild type or A380 mutant of PPM1D, and were stained with TRF2 and TIN2 antibodies. Relative TIN2 intensity in TRF2 foci is plotted ± SD, *n* ≥ 286. Statistical significance was evaluated using Mann–Whitney test (****P < 0.0001). (**B**) Plotted is the mean intensity of TRF2 staining in nuclear foci ± SD in cells from K. Statistical significance was evaluated using Mann–Whitney test (*P < 0.05, ****P < 0.0001), *n* ≥ 286. The scale bar in representative images corresponds to 10 μm. (**C**) Expression of the catalytic domain of PPM1D was induced or not in RPE1-PPM1D-A380 cells by addition of doxycycline for 10 days and where indicated, PPM1D inhibitor was added to the media 1 h prior fixation. Cells were hybridized with TAACCC FISH-probe, stained for 53BP1, and formation of TIFs was quantified by ScanR microscopy. Plotted is a fraction of cells with more than three TIFs. Mean ± SD is shown, statistical significance was evaluated by unpaired *t*-test (*n* = 4). Whole cell lysates were evaluated by immunoblotting, phosphorylation of KAP1 at S824 is a marker of ATM activity, TurboGFP is a marker of PPM1D-A380 expression, the asterisk indicates a non-specific band. Note that PPM1D (Santa Cruz) recognizes only the endogenous full length PPM1D. (**D**) Quantification of chromosome fusions in RPE1-PPM1D-A380 cells treated or not with doxycycline for 10 days. More than 1246 chromosomes per condition was analyzed in each of the three independent experiments. Mean ± SD is shown, statistical significance was evaluated by paired *t*-test. The scale bars in the representative images corresponds to 10 or 2 μm, respectively. (**E**) HeLa cells with tetracycline-inducible knock down of endogenous TRF2 were stably reconstituted with the wild type or S410A mutant GFP-TRF2 and single cell clones were cultured in the absence or presence of doxycycline for 5 days. Whole cell lysates were analyzed by immunoblotting. (**F**) Cells from E were seeded into 96 wells at 100 cells/well, and cultured for additional 7 days. Relative cell proliferation was determined by resazurin assay. Statistical significance was determined by unpaired *t*-test, *n* = 3.

As the interaction of TRF2 and TIN2 responded to the inhibition of PPM1D, we asked if increased activity of PPM1D might interfere with function of the shelterin complex at telomeres. Indeed, we found that overexpression of the wild-type PPM1D decreased the amount of TIN2 at telomeres (Figure 5A). In addition, we observed that expression of the A380 fragment of PPM1D (that showed the strongest targeting to the telomeres in Figure 2E) efficiently stripped TIN2 from the telomeres (Figure 5A). Of note, expression of the A380 fragment of PPM1D also reduced the intensity of TRF2 staining at telomeres suggesting that assembly of the shelterin may be impaired after dephosphorylation by PPM1D (Figure 5B).

To study consequences of the increased PPM1D expression, we developed a doxycycline-inducible RPE1-PPM1D-A380 cells (Supplementary Figure S4A), and followed formation of TIFs upon treatment with doxycycline for 10 days (Figure 5C). Although the fraction of cells with > 3 TIFs was slightly higher in cells treated with doxycycline compared to control cells, the difference was not statistically significant (Figure 5C). As PPM1D can target γH2AX and ATM, we hypothesised that failure to form TIFs could be caused by overall suppression of DDR by PPM1D activity (8,74). Therefore, we treated RPE1-PPM1D-A380 cells for 10 days to allow formation of potential telomeric damage and then treated cells with PPM1D inhibitor just before fixation to allow activation of DDR. Indeed, transient PPM1D inhibition increased activity of ATM as documented by increased level of KAP1-S824 phosphorylation (Figure 5C). Consistently, upon transient inhibition of PPM1D, we observed a significant increase of TIF formation in cells expressing PPM1D-A380 suggesting that these cells experienced telomeric damage (Figure 5C). Next, we analyzed telomeric damage in RPE1-PPM1D-A380 cells by telomeric FISH in metaphase spreads (Figure 5D). We noted that the fraction of telomeric fusions was doubled in RPE1-PPM1D-A380 cells treated with doxycycline compared to control cells Figure 5D) confirming that increased PPM1D activity in cells promotes damage of the telomeric DNA.

Finally, we asked if the phosphorylation of TRF2 is required for cell proliferation. To this end, we used HeLa cells with inducible knock down of endogenous TRF2 and stably reconstituted them with the wild-type or S410A mutant TRF2 (Figure 5E). After 12 days of doxycycline treatment, we compared relative proliferation and found that two independent clones expressing S410A TRF2 proliferated significantly worse than the cells expressing the wild-type TRF2 (Figure 5F) suggesting that impaired phosphorylation of TRF2 leads to suppression of cell proliferation.

### Loss of PPM1D supresses recruitment of DNA repair proteins to the DSBs at telomeres

**Figure 6.**
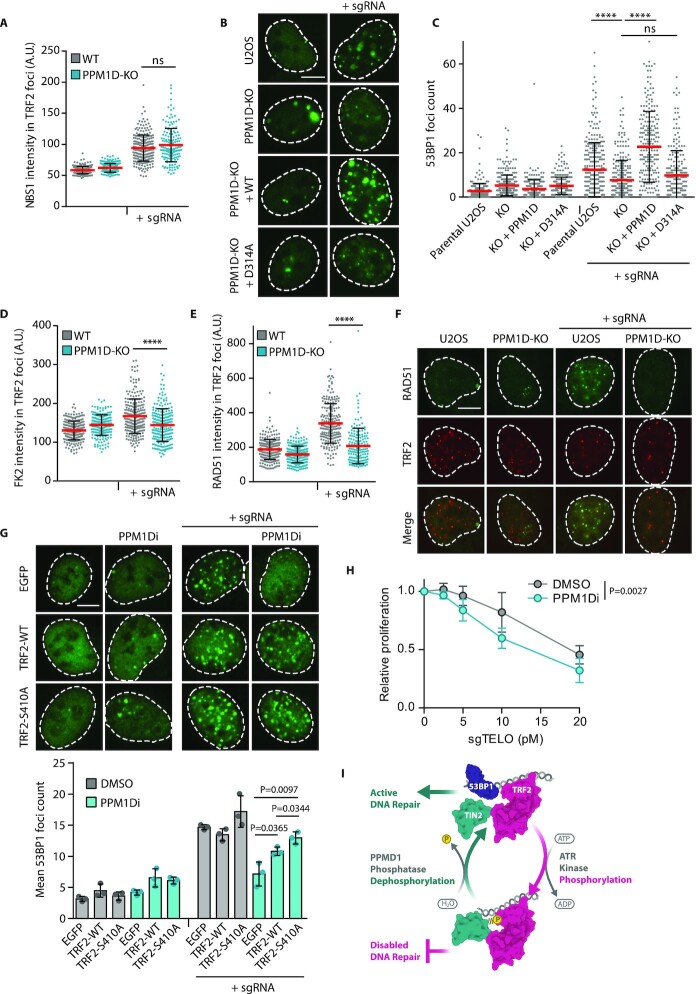
Loss of PPM1D affects recruitment of DNA repair factors to telomeric breaks. (**A**) Parental and U2OS-PPM1D-KO cells were transfected with plasmids coding for Cas9-EGFP with or without the telomere-targeting sgRNA. After 24 h, cells were fixed and stained for NBS1 and TRF2. Plotted is the mean NBS1 signal in TRF2 foci ± SD, *n* ≥ 171. Statistical significance was evaluated using Mann–Whitney test. Representative experiment is shown from two independent repeats. (**B**) Parental, U2OS-PPM1D-KO cells and U2OS-PPM1D-KO cells stably expressing FLAG-PPM1D variants were transfected with plasmids coding for Cas9-EGFP with or without the telomere-targeting sgRNA. After 24 h, cells were fixed and stained for 53BP1, the scale bar represents 10 μm. (**C**) Quantification of (B). Plotted is the mean of 53BP1 foci count ± SD, *n* ≥ 221. Statistical significance was evaluated using Mann–Whitney test (****P < 0.0001). Representative experiment is shown. (**D**) Cells were treated as in A and were stained for TRF2 and conjugated ubiquitin using Fk2 antibody. Plotted is the mean FK2 signal in TRF2 foci ± SD, *n* ≥ 205. Statistical significance was evaluated using Mann–Whitney test (****P < 0.0001). Representative experiment is shown from two independent repeats. (**E**) Parental and U2OS-PPM1D-KO cells were transfected as in (A), fixed, and stained for RAD51 and TRF2. Plotted is mean RAD51 intensity in TRF2 foci ± SD, *n* ≥ 161. Statistical significance was evaluated using Mann–Whitney test (****P < 0.0001). Representative experiment is shown from two independent repeats. (**F**) Representative images for (E), the scale bar represents 10 μm. (**G**) Parental and U2OS-PPM1D-KO cells were co-transfected with plasmids coding for GFP or GFP-TRF2 variants, and FLAG-Cas9 with or without the telomere-targeting sgRNA, and treated or not with PPM1Di for 24 h. Cells were fixed and stained for 53BP1 and FLAG. Only FLAG and GFP double positive cells were analyzed. Means of three independent experiments are plotted ± SD. Statistical significance was evaluated using unpaired *t*-test. Representative images are shown, the scale bar represents 10 μm. (**H**) RPE1-iCut cells were treated overnight with doxycycline and Shield-1 and telomeric DNA damage was induced by transfection of indicated amounts of telomeric sgRNA. Cells were incubated with DMSO or PPM1D inhibitor for 7 days. Relative proliferation was determined by resazurin assay and was normalized to non-treated cells (*n* = 3). (**I**) Model of pTRF2-S410 function at telomere. Under basal conditions, non-phosphorylated TRF2 interacts with TIN2 through its TRFH domain and with telomeric DNA through its Myb domain. Induction of DSBs at telomeres leads to recruitment of DNA repair factors including 53BP1. Upon activation of ATR, TRF2 is phosphorylated at S410, which promotes tight binding of TIN2 and protects the broken telomere from recruitment of 53BP1. Loss of PPM1D activity leads to hyper-phosphorylation of TRF2 and prevents recruitment of 53BP1 to the telomeric DSBs, possibly decreasing the risk of the telomere fusion.

Finally, we investigated the consequence of altered PPM1D activity for DNA repair at telomeres. We induced DSBs at telomeres by Cas9 and compared recruitment of various DNA repair factors in control cells and in PPM1D-KO cells. We found no difference in recruitment of NBS1 suggesting that recognition of the DNA break by MRN complex was unaffected by the loss of PPM1D (Figure 6A, Supplementary Figure S4B). In contrast, we observed that recruitment of 53BP1 protein to telomeric DSBs was significantly reduced in U2OS-PPM1D-KO cells (Figure 6B, C). Similarly, formation of the 53BP1 foci upon Cas9-mediated DNA damage at telomeres was impaired in MCF7 and RPE1 cells treated with PPM1D inhibitor (Supplementary Figure S4C, D). Importantly, recruitment of 53BP1 to damaged telomeres was rescued in U2OS-PPM1D-KO cells by expression of the wild type EGFP-PPM1D (but not with the phosphatase dead D314A mutant) confirming that the phenotype was indeed caused by a loss of PPM1D activity (Figure 6B, C). We also noted that the level of protein ubiquitination detected by FK2 antibody was reduced at damaged telomeres in U2OS-PPM1D-KO cells (Figure 6D, Supplementary Figure S4E). Histone H2A ubiquitination is required for recruitment of 53BP1 and BRCA1 to DNA damage foci, and thus the lack of ubiquitination at telomeres may explain the decreased level of 53BP1 in cells treated with PPM1D inhibitor (75). As the mouse TRF2 has previously been shown to recruit a deubiquitinating enzyme BRCC3 through a so-called iDDR region (36), we tested if the observed defect of 53BP1 binding upon inhibition of PPM1D could be rescued by depletion of BRCC3. However, we did not observe any difference in 53BP1 recruitment to the telomeric DSBs suggesting that the phosphorylation of TRF2 at S410 suppresses 53BP1 recruitment through a distinct molecular mechanism than the iDDR region (Supplementary Figure S4F).

Besides impaired formation of 53BP1 foci, we also observed strongly reduced recruitment of RAD51 to the telomeric breaks suggesting that the repair through homologous recombination is also impaired (Figure 6E, F). To investigate if the effect of PPM1D inhibition on TRF2 phosphorylation and reduced recruitment of 53BP1 are functionally linked, we co-expressed Cas9 together with the telomeric sgRNA and various forms of TRF2 in cells treated or not with PPM1D inhibitor. Whereas expression of the wild-type EGFP-TRF2 did not fully rescue recruitment of 53BP1 to damaged telomeres, expression of the EGFP-TRF2-S410A mutant significantly increased the level of 53BP1 at damaged telomeres (Figure 6G). This result suggests that PPM1D promotes recruitment of 53BP1 to DNA breaks at telomeres by dephosphorylating TRF2.

To evaluate the functional outcome of PPM1D inhibition at damaged telomeres, we determined the relative proliferation of RPE1-iCut cells upon induction of a mild telomeric DNA damage achieved by titrating down of the amount of telomeric sgRNA (46) (Figure 6H, Supplementary Figure S4G). We found that PPM1D inhibition significantly suppressed proliferation of the RPE1-iCut cells that experienced telomeric DNA damage (Figure 6H). We conclude that PPM1D activity is needed for the cell response to telomeric DNA damage although the precise molecular defect in DNA repair remains to be addressed by future research.

In the summary, we show that TRF2 is phosphorylated at S410 upon DNA damage at telomeres by ATR which promotes its interaction with TIN2 and limits recruitment of 53BP1 to the breaks. Phosphorylation of TRF2 is reversed by the activity of PPM1D phosphatase that promotes recruitment of 53BP1 to telomeres (Figure 6I). Physiological levels of TRF2 phosphorylation are required for cell survival as increased TRF2 phosphorylation does not allow efficient repair, while impaired TRF2 phosphorylation supresses shelterin complex assembly and may lead to telomeric fusions.

## DISCUSSION

Several components of the shelterin complex were reported to undergo phosphorylation at various conditions, however only some of these events were thoroughly characterized (76). Most importantly, CDK-dependent phosphorylation of TRF2 at Ser365 prevents recruitment of the helicase RTEL1 to telomeres (35). During S phase, TRF2-Ser365 is dephosphorylated by PP6 phosphatase that promotes recruitment of RTEL1, unwinding the t-loops and telomere replication (34,35). Following exposure of cells to ionizing radiation, TRF2 was reported to be transiently phosphorylated at Thr230 allowing its association with DNA lesions outside the telomeres and promoting DNA repair (77–79). However, the role of TRF2 modification in DNA repair of the telomeric lesions has remained unclear.

Here, we report a new phosphorylation of TRF2 at S410 that is strongly induced by Cas9-mediated DSBs at telomeres. Using specific small-molecule inhibitors and RNA interference, we identify ATR as the major kinase responsible for TRF2-S410 modification at damaged telomeres. Further, we show that the level of TRF2-S410 phosphorylation is tightly regulated by PPM1D phosphatase that associates with TRF2 and localizes at the telomeres. Loss of PPM1D or inhibition of its enzymatic activity strongly induced TRF2-S410 phosphorylation at telomeres and promoted recruitment of TIN2 and TPP1 to the telomeres. Since the S410 is located close to the TBM2 region responsible for the interaction with TIN2, we tested the impact of TRF2-S410 phosphorylation on this interaction. An unbiased proteomic approach revealed that the phosphorylated peptide spanning residues 403–417 of TRF2 (but not the non-phosphorylated counterpart), pulled down the TIN2-TPP1-POT1 trimer from the nuclear extract. Subsequently, a fluorescence anisotropy assay performed with synthetic peptides and with purified TIN2 confirmed that TRF2 phosphorylation at S410 increases the affinity between TRF2 and TIN2. When expressed in cells, the non-phosphorylatable TRF2-S410A mutant was able to interact with TIN2, which suggests that phosphorylation is not critically needed for mediating the interaction. On the other hand, the PLA assay revealed a stronger interaction between TRF2 and TIN2 upon inhibition of PPM1D that increases the level of TRF2 phosphorylation at S410. As TRF2 and TIN2 protect the ends of telomeres by promoting t-loop formation, we tested if the activity of PPM1D affects the architecture of the telomeric ends through regulating the shelterin complex assembly. To address this, we imaged the telomeres in psoralen-crosslinked chromatin spreads using Structured Illumination Microscopy and determined the fractions of linear and closed telomeres. Consistent with the published literature, we observed t-loops in about 25% of telomeres in parental cells (33). Nevertheless, fraction of the t-loops was comparable in U2OS-PPM1D-KO cells suggesting that PPM1D activity may not affect the t-loop formation. As approximately half of the imaged telomeres is excluded from the analysis due to inconclusive shape, we also cannot rule out the possibility that the assay is not sensitive enough to detect mild differences in the t-loop formation. Alternatively, the activity of PPM1D may impact a higher-order organization of the telomeres mediated in cis and trans by TRF2 and TIN2 (40).

The main finding of this study is that PPM1D is needed for DNA damage response at telomeric DSBs (Figure 6I). When PPM1D activity was present, cells recruited DNA repair factors to the DSBs located at telomeres. Conversely, loss or inhibition of PPM1D impaired recruitment of the DNA repair factors 53BP1 and RAD51 to the broken telomeres. As the non-phosphorylatable TRF2-S410A mutant rescued the recruitment of 53BP1 significantly better than the wild-type TRF2, we concluded that phosphorylation of TRF2 inhibits DNA damage response at telomeres. The dimerization domain and the iDDR region (corresponding to residues 449–473 of human TRF2) within the hinge domain of TRF2 were previously shown to supress the DNA damage response at telomeres by preventing activation of ATM and by inhibiting the RNF168-dependent ubiquitination, respectively (36). We found that the formation of 53BP1 foci at telomeric DSBs was not rescued by depletion of the BRCC3 or UBR5 in U2OS-PPM1D-KO cells suggesting that PPM1D affects DDR independently of the iDDR region in TRF2. We hypothesize that DSB-induced phosphorylation of TRF2 may allow cells to re-establish the telomere organization by promoting TRF2 association with TIN2-TPP1-POT1. An increased assembly of the shelterin may then interfere with the recruitment of 53BP1 to the break, thus limiting the risk of telomeric fusions. In contrast, dephosphorylation of TRF2 and weakening its interaction with TIN2-TPP1-POT1 could make the telomere more accessible to the recruitment of the DNA repair proteins.

We also noted that overexpression of PPM1D decreased the levels of TRF2 at telomeres which is in line with the disassembly of the shelterin after dephosphorylation of its components. However, we did not observe the formation of the TIFs upon overexpression of PPM1D, possibly due to the ability of PPM1D to efficiently suppress the activity of ATM (7,80). We propose that PPM1D activity needs to be tightly balanced at telomeres to allow the recruitment of DNA repair proteins to DSBs while preventing disassembly of the shelterin from the telomeres. Of note, high levels of active PPM1D are commonly present in cancer cells due to amplification of the chromosomal locus 17q23 or due to gain-of-function mutations in the last exon of *PPM1D* (11,12,65,81,82). It is tempting to speculate that besides the established role of the overexpressed PPM1D in overriding the cell cycle checkpoint, the increased activity of PPM1D could promote genome instability in cancer cells by interfering with the telomere functions.

## Supplementary Material

gkac1269_Supplemental_Files

## Data Availability

Data from the mass spectrometry analysis were uploaded to the PRIDE database (accession numbers PXD035268 and PXD035273) and are also provided as source data in the supplementary material.
