# Supplementary material for: Phosphorylation of TRF2 promotes its interaction with TIN2 and regulates DNA damage response at telomeres
Source: Nucleic Acids Res. 2023 Jan 18;51(3):1154–72. doi: 10.1093/nar/gkac1269 (PMC9943673; doi:10.1093/nar/gkac1269)
Supplement: gkac1269_Supplemental_Files [file gkac1269_supplemental_files.zip › Supplemetary data-figures-legends-combined.pdf]

SUPPLEMENTARY DATA

Phosphorylation of TRF2 promotes its interaction with TIN2 and regulates DNA damage response at telomeres

Radka Storchova[1,#], Matous Palek[1,#], Natalie Palkova[1], Pavel Veverka[2], Tomas Brom[2], Ctirad Hofr[2], Libor Macurek[1]

[1] *Cancer Cell Biology, Institute of Molecular Genetics of the Czech Academy of Sciences, Prague, CZ-14220, Czech Republic*

[2] *LifeB, Functional Genomics and Proteomics, National Centre for Biomolecular Research, Faculty of Science, Masaryk University, Brno, CZ-62500, Czech Republic*

Suppl. Fig. 1. PPM1D interacts with TRF2 in cells and *in vitro*

Suppl. Fig. 2. PPM1D dephosphorylates TRF2

Suppl. Fig. 3. PPM1D activity controls interaction between TRF2 and TIN2

Suppl. Fig. 4. PPM1D activity is needed for recruitment of DNA repair factors to telomeres

Supplementary Table 1. Identification of proteins interacting with PPM1D by proximity biotinylation

Supplementary Table 2. Identification of proteins interacting with pTRF2-S410 peptide

Suppl. Fig. 1. PPM1D interacts with TRF2 in cells and *in vitro*

A. EGFP or EGFP-TRF2 were isolated from transfected HEK293 cells in the presence of high salt using GFP trap. Beads with bound proteins were washed with PBS and were incubated with purified His-PPM1D. After three washing with PBS, bound PPM1D was analyzed by immunoblotting.

B. U2OS cells were treated with DMSO or with PPM1D inhibitor and after fixation, PLA assay was performed using two distinct sets of antibodies. Plotted is the count of nuclear PLA foci, bars indicate mean ±SD, n=300. Statistical significance was evaluated using Mann–Whitney test, (****P<0.0001). Representative experiment is shown from two independent repeats. The scale bar in representative images corresponds to 10 µm.

C. MCF7 cells transfected with control siRNA (siNC) or siRNA to TRF2 and were treated or not with PPM1D inhibitor. Whole cell lysates were probed with indicated antibodies.

D. Validation of TRF2 antibody in immunoblotting. Cells were transfected twice with control siRNA (siNC), siRNA to TRF1 or two various siRNAs to TRF2. Six days after transfection, cells were lysed and proteins were analyzed by immunoblotting.

E. Validation of TRF2 antibody in immunofluorescence. Cells were treated as in D and after fixation they were probed with a mouse or rabbit antibody against TRF2. Plotted is the mean nuclear intensity ±SD, n=300. Statistical significance was evaluated using Mann–Whitney test (****P<0.0001). Representative experiment is shown from two independent repeats. The scale bar in representative images corresponds to 10 µm.

F. Localization of PPM1D and its deletion mutants. Cells transfected with the wild-type EGFP-PPM1D, -PPM1D-A380, -PPM1D-ΔPro, -PPM1D-CT, -PPM1D-ΔB mutant or empty EGFP plasmid were probed with TRF2 antibody and imaged by ScanR microscopy. Representative images are shown, GFP signal intensity is visualised using fire look up table. The scale bars represents 10 µm and 2 µm, respectively.

G. Quantification of E. Plotted is the relative mean EGFP intensity in TRF2 positive foci, the red line indicates mean ±SD, n≥292. Statistical significance was evaluated using Mann–Whitney test, (****P < 0.0001). Representative experiment is shown from two independent repeats.

H. Sequence alignment of TRF2 from different species. The green rounded rectangle represents a conserved helix in TBM2.

I. Parental U2OS or U2OS-PPM1D-KO cells were transfected with plasmids coding for Cas9-EGFP with or without telomeric repeat-targeting sgRNA. After 24 h, cells were fixed and stained for TRF2 and γH2AX, scale bar represents 10 µm. Representative images from the quantification shown in Fig. 3G.

Suppl. Fig. 1

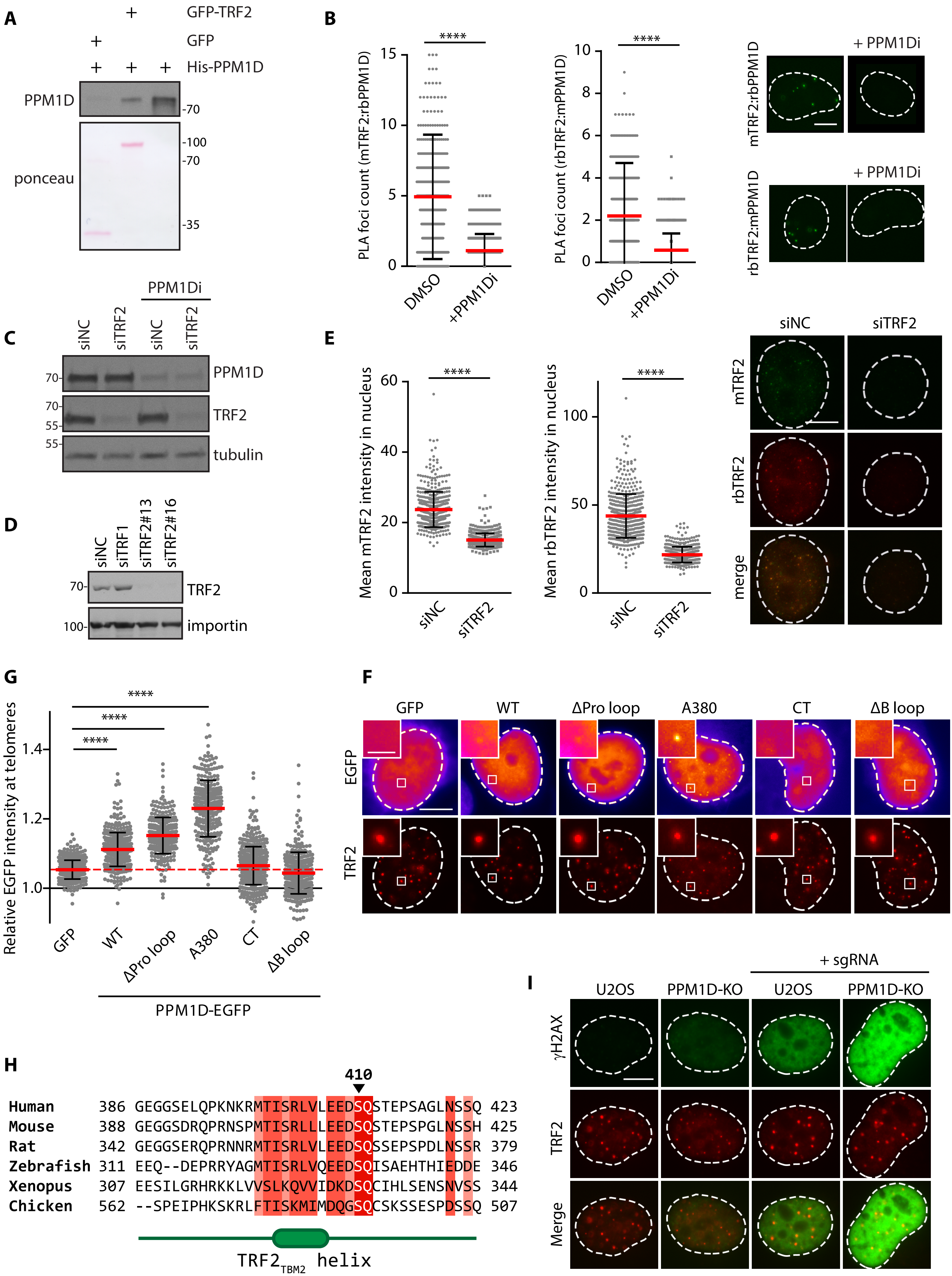
A
GFP-TRF2
GFP
His-PPM1D
PPM1D
ponceau
-70
-100
-70
-35
B
PLA foci count (mTRF2:rbPPM1D)
PLA foci count (rbTRF2:mPPM1D)
****
DMSO
+PPM1Di
mTRF2:rbPPM1D
rbTRF2:mPPM1D
+ PPM1Di
C
PPM1Di
siNC
siTRF2
PPM1D
TRF2
tubulin
D
siNC
siTRF1
siTRF2#13
siTRF2#16
TRF2
importin
E
Mean mTRF2 intensity in nucleus
Mean rbTRF2 intensity in nucleus
siNC
siTRF2
mTRF2
rbTRF2
merge
G
Relative EGFP intensity at telomeres
GFP
WT
ΔPro loop
A380
CT
ΔB loop
PPM1D-EGFP
F
EGFP
TRF2
H
410
Human 386 GEGGSELQPKNKRMTISRLVLEEDSQSTEPSAGLNSSQ 423
Mouse 388 GEGGSDRQPRNSPMTISRLLLEEDSQSTEPSPGLNSSH 425
Rat 342 GEGGSERQPRNNRMTISRLVLEEDSQSSEPSPDLNSSR 379
Zebrafish 311 EEQ--DEPRRYAGMTISRLVQEEDSQISAEHTHIEDDE 346
Xenopus 307 EESILGRHRKKLVVSLKQVVIDKDSQCIHLSENSNVSS 344
Chicken 562 --SPEIPHKSKRLFTISKMIMDQGSQCSKSSESPDSSQ 507
TRF2 TBM2 helix
I
+ sgRNA
U2OS
PPM1D-KO
γH2AX
TRF2
Merge

Suppl. Fig. 2. PPM1D dephosphorylates TRF2

A. Parental U2OS or U2OS-PPM1D-KO cells were transfected with plasmids coding for Cas9-EGFP with or without telomeric repeat-targeting sgRNA. Cells were fixed at indicated time points, hybridized with TAACCC FISH-probe, and analyzed by ScanR microscopy. Representative images are shown, the scale bar corresponds to 10 µm.

B. Quantification from A. Plotted is the mean area of telomeres, the mean ±SD is shown, n=300. Statistical significance was evaluated using Mann–Whitney test.

C. Quantification from A. Plotted is telomere count, the mean ±SD is shown, n=300. Statistical significance was evaluated using Mann–Whitney test.

D. Parental U2OS, U2OS-PPM1D-KO cells and U2OS-PPM1D-KO cells stably transfected with FLAG-PPM1D were transfected with plasmids coding for Cas9-EGFP with or without telomeric repeat-targeting sgRNA. After fixation, cells were probed with pTRF2-S410 and TRF2 antibodies. The scale bar represents 10 µm. Representative image from the quantification shown in Fig. 3I.

E. U2OS cells were transfected with plasmids coding for Cas9-EGFP with or without telomeric repeat-targeting sgRNA. Upon transfection, cells were treated with DMSO or inhibitors of ATM, ATR or DNA-PK for 20 h. After fixation, cells were probed with pTRF2-S410 and TRF2 antibodies, the scale bar represents 10 µm. Representative images from the quantification shown in Fig. 3K.

F. U2OS cells were transfected with indicated siRNAs. Next day, cells were transfected with plasmids coding for Cas9-EGFP with or without the telomere-targeting sgRNA. Cells were fixed 24h after transfection, stained with TRF2 and pTRF2-S410 antibodies, and analysed using ScanR microscopy. Representative images are shown, the scale bar corresponds to 10 µm.

G. Quantification form F, Plotted is the mean pTRF2-S410 intensity in TRF2 foci, the mean ±SD is shown, $n \geq 246$. Statistical significance was evaluated using Mann–Whitney test (****$P < 0.0001$).

H. Depletion of ATM and ATR in F was evaluated by immunoblotting.

Suppl. Fig. 2

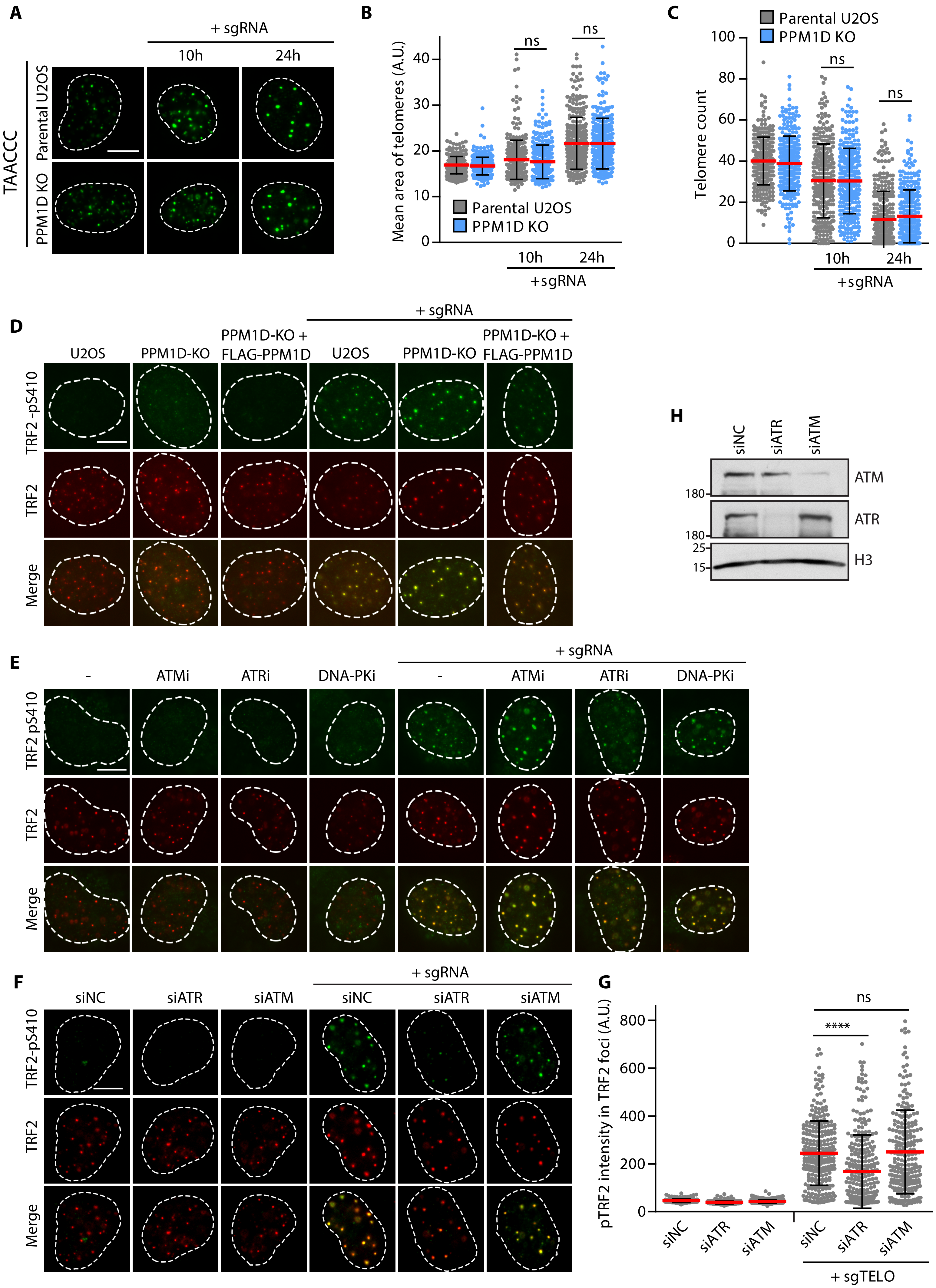
A
+ sgRNA
10h
24h
TAACCC
Parental U2OS
PPM1D KO
B
Mean area of telomeres (A.U.)
ns
Parental U2OS
PPM1D KO
10h
24h
+sgRNA
C
Telomere count
Parental U2OS
PPM1D KO
ns
10h
24h
+sgRNA
D
+ sgRNA
U2OS
PPM1D-KO
PPM1D-KO + FLAG-PPM1D
TRF2 -pS410
TRF2
Merge
H
siNC
siATR
siATM
ATM
ATR
H3
180
25
15
E
+ sgRNA
-
ATMi
ATRi
DNA-PKi
TRF2 pS410
TRF2
Merge
F
+ sgRNA
siNC
siATR
siATM
TRF2-pS410
TRF2
Merge
G
pTRF2 intensity in TRF2 foci (A.U.)
ns
****
siNC
siATR
siATM
+ sgTELO

Suppl. Fig. 3. PPM1D activity controls interaction between TRF2 and TIN2

A. Alphafold Colab was used to model interaction of TIN2 (residues 1-158) and TRF2 (residues 392-420). Superimposition of Alphafold model with experimentally determined structure (PDB ID: 5XYF, semitransparent) is shown on left. Alphafold model on the right shows the interaction interface between $TRF2_{TBM2}$ and $TIN2_{TRFH}$. The S410 at the extension of $TRF2_{TBM2}$ α-helix is oriented opposite basic residues of $TIN2_{TRFH}$ (R50, R52, H53, R56). Semitransparent representation of TIN2 surface is colored by electrostatic potential that was determined using APBS Electrostatics Plugin in PyMOL (positive potential in blue, negative potential in red).

B. Validation of TIN2 antibody in immunofluorescence. Cells were transfected with control siRNA (siNC), or siRNA to TIN2. Four days after transfection, cells were fixed and probed with indicated combinations of TIN2 and TRF2 antibodies (mTIN2 mouse-anti-TIN2; rbTIN2 rabbit-anti-TIN2; mTRF2 mouse-anti-TRF2, rbTRF2 rabbit-anti-TRF2). Plotted is the mean TIN2 intensity in TRF2 foci ±SD, n=300. Statistical significance was evaluated using Mann–Whitney test (****P<0.0001). The scale bar in representative images corresponds to 10 µm.

C. MCF7 cells were treated with DMSO or with PPM1D inhibitor for 24 h, fixed and probed with TPP1 and TRF2 antibodies, n=350. Mean TPP1 intensity in TRF2 foci normalized to the mean nuclear TPP1 intensity is plotted ±SD. Statistical significance was evaluated using Mann–Whitney test (****P < 0.0001). The scale bar in representative images corresponds to 10 µm.

D. MCF7 cells were treated as in C and probed with pTRF2-S410 and TRF2 antibodies. Mean pTRF2-S410 intensity in TRF2 foci normalized to the mean nuclear pTRF2-S410 intensity is plotted ±SD. Shown is quantification of 500 cells. Statistical significance was evaluated using Mann–Whitney test (****P < 0.0001). The scale bar in representative images corresponds to 10 µm.

E. MCF7 cells were treated as in C and probed with TIN2 and TRF2 antibodies. Mean TIN2 intensity in TRF2 foci normalized to the mean nuclear TIN2 intensity is plotted ±SD, n=500. Statistical significance was evaluated using Mann–Whitney test (****P < 0.0001). The scale bar in representative images corresponds to 10 µm.

F. Chromosome spreads from parental U2OS and U2OS-PPM1D-KO cells were hybridized with TAACCC FISH-probe and imaged by 3D-SIM. Representative images are shown. Bars indicate 10 µm and 1 µm respectively.

Suppl. Fig. 3

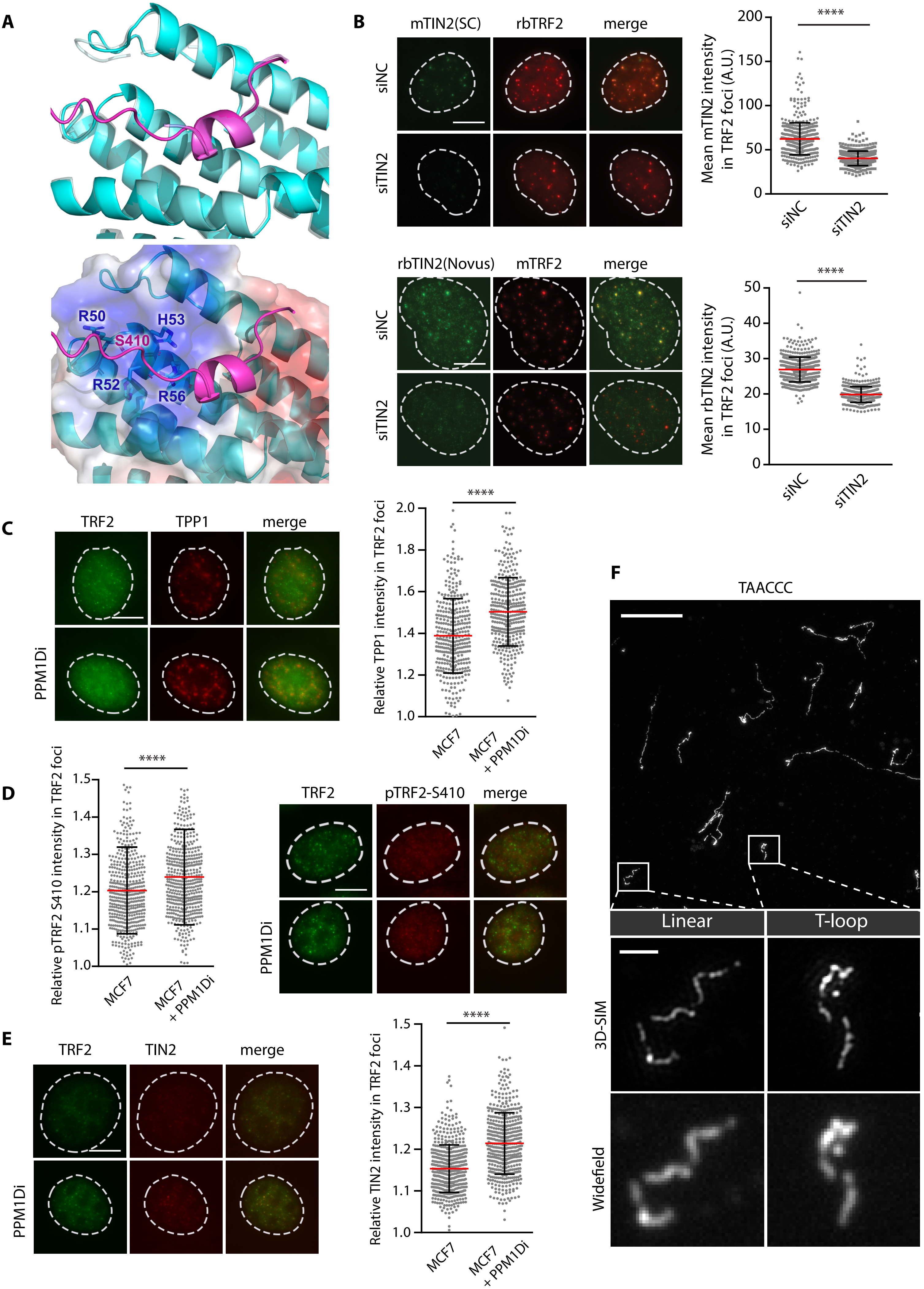

A
R50
H53
S410
R52
R56
B
mTIN2(SC)
rbTRF2
merge
siNC
siTIN2
Mean mTIN2 intensity in TRF2 foci (A.U.)
****
rbTIN2(Novus)
mTRF2
merge
Mean rbTIN2 intensity in TRF2 foci (A.U.)
C
TRF2
TPP1
merge
PPM1Di
Relative TPP1 intensity in TRF2 foci
MCF7
MCF7 + PPM1Di
D
Relative pTRF2 S410 intensity in TRF2 foci
TRF2
pTRF2-S410
merge
E
TRF2
TIN2
merge
Relative TIN2 intensity in TRF2 foci
F
TAACCC
Linear
T-loop
3D-SIM
Widefield

Suppl. Fig. 4. PPM1D activity is needed for recruitment of DNA repair factors to telomeres

A. RPE1 cells stably transfected with plasmid for doxycycline-inducible expression of active PPM1D A380 fragment and a marker turboGFP from the same promoter were cultivated in the absence or presence of doxycycline for 48 h. Whole cell lysates were analyzed by immunoblotting. Note that PPM1D antibody (D4F7) recognizes an epitope within the catalytic domain of PPM1D and thus reacts with the A380 fragment of PPM1D migrating at 50 kDa.

B. Parental and U2OS-PPM1D-KO cells were transfected with plasmids coding for Cas9-EGFP with or without the telomere-targeting sgRNA. After 24 h, cells were fixed and stained for TRF2 and NBS1 antibody. Representative images are shown, the scale bar represents 10 µm. Quantification is shown in Fig. 6A.

C. MCF7 cells were transfected with plasmids coding for Cas9-EGFP with or without the telomere-targeting sgRNA, and treated or not with PPM1Di for 24 h. Cells were fixed and stained for 53BP1. Representative images are shown (lower panel), the scale bar represents 10 µm. Plotted is the mean of 53BP1 foci count ±SD, n≥188 (upper panel). Statistical significance was evaluated using Mann–Whitney test (****$P < 0.0001$).

D. RPE1 cells were treated and analyzed as in C.

E. Parental and U2OS-PPM1D-KO cells were transfected with plasmids coding for Cas9-EGFP with or without the telomere-targeting sgRNA. After 24 h, cells were fixed and stained for TRF2 and conjugated ubiquitin by FK2 antibody. Representative images are shown, scale bar represents 10 µm. Quantification is shown in Fig. 6D.

F. U2OS cells were transfected with indicated siRNAs. Subsequently, cells were transfected with plasmids coding for Cas9-EGFP with or without the telomere-targeting sgRNA and formation of 53BP1 foci was assayed in EGFP positive cells by ScanR microscopy. Mean ±SD is shown, N=3.). Statistical significance was evaluated using unpaired t-test (**$P < 0.01$). Depletion of BRCC3 was evaluated by immunoblotting.

G. RPE1-iCut cells were treated overnight with doxycycline and Shield-1. Next day, they were transfected with telomeric repeats targeting sgRNA. Cells were fixed 7h after transfection, stained with TRF2 and 53BP1 antibody and imaged by ScanR.

Suppl. Fig. 4

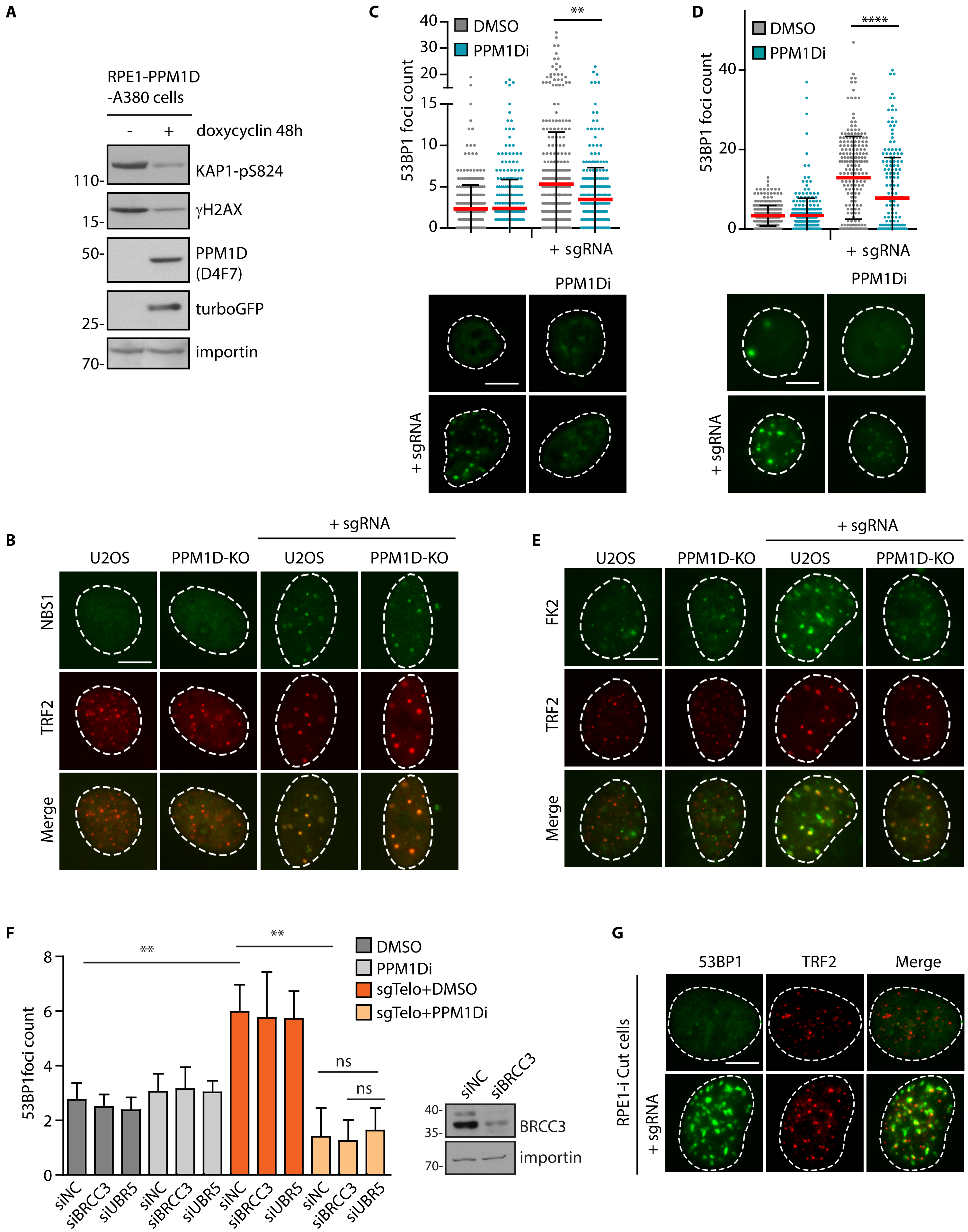
A
RPE1-PPM1D
-A380 cells
- + doxycyclin 48h
KAP1-pS824
110-
γH2AX
15-
50-
PPM1D
(D4F7)
turboGFP
25-
importin
70-
C
53BP1 foci count
DMSO
PPM1Di
**
+ sgRNA
PPM1Di
+ sgRNA
D
53BP1 foci count
DMSO
PPM1Di
****
+ sgRNA
PPM1Di
+ sgRNA
B
+ sgRNA
U2OS
PPM1D-KO
U2OS
PPM1D-KO
NBS1
TRF2
Merge
E
+ sgRNA
U2OS
PPM1D-KO
U2OS
PPM1D-KO
FK2
TRF2
Merge
F
53BP1foci count
**
**
ns
ns
DMSO
PPM1Di
sgTelo+DMSO
sgTelo+PPM1Di
siNC
siBRCC3
siUBR5
siNC
siBRCC3
siUBR5
siNC
siBRCC3
siUBR5
siNC
siBRCC3
siUBR5
siNC
siBRCC3
40-
35-
BRCC3
70-
importin
G
53BP1
TRF2
Merge
RPE1-i Cut cells
+ sgRNA

Supplementary Table 1. Identification of proteins interacting with PPM1D by proximity biotinylation

HEK293 stably expressing empty pBIOID2 or pBIOID2-PPM1D-D314A were treated with biotin and lysed under denaturating conditions. Biotinylated proteins were pulled-down by streptavidin beads, digested by trypsin and peptides were identified by LC/MS analysis. The data were submitted to MaxQuant and analyzed using Perseus software. Table shows the measured LFQ values from three biological replicates. The $-\log_{10}$ (P value) was calculated by t-test and hits with FDR<0.05 were considered significant.

Supplementary Table 2. Identification of proteins interacting with pTRF2-S410 peptide

Biotinylated non-phosphorylated and phosphorylated TRF2 peptides were incubated with nuclear extracts and pulled down by streptavidin beads. After digestion with trypsin, peptides were identified by LC/MS analysis. The data were submitted to MaxQuant and analyzed using Perseus software. Table shows the measured LFQ values from three biological replicates. The $-\log_{10}$ (P value) was calculated by t-test and hits with FDR<0.1 were considered significant.
